# DECA: harnessing interpretable transformer model for cellular deconvolution of chromatin accessibility profile

**DOI:** 10.1093/bib/bbaf069

**Published:** 2025-02-23

**Authors:** Shijie Luo, Ming Zhu, Liquan Lin, Jiajing Xie, Shihao Lin, Ying Chen, Jiali Zhu, Jialiang Huang

**Affiliations:** State Key Laboratory of Cellular Stress Biology, School of Life Sciences, Faculty of Medicine and Life Sciences, Xiamen University, No. 4221, Xiang'an South Road, Xiamen, Fujian 361102, China; National Institute for Data Science in Health and Medicine, Xiamen University, No. 4221, Xiang'an South Road, Xiamen, Fujian 361102, China; State Key Laboratory of Cellular Stress Biology, School of Life Sciences, Faculty of Medicine and Life Sciences, Xiamen University, No. 4221, Xiang'an South Road, Xiamen, Fujian 361102, China; State Key Laboratory of Cellular Stress Biology, School of Life Sciences, Faculty of Medicine and Life Sciences, Xiamen University, No. 4221, Xiang'an South Road, Xiamen, Fujian 361102, China; National Institute for Data Science in Health and Medicine, Xiamen University, No. 4221, Xiang'an South Road, Xiamen, Fujian 361102, China; State Key Laboratory of Cellular Stress Biology, School of Life Sciences, Faculty of Medicine and Life Sciences, Xiamen University, No. 4221, Xiang'an South Road, Xiamen, Fujian 361102, China; School of Informatics, Xiamen University, No. 4221, Xiang'an South Road, Fujian 361000, China; State Key Laboratory of Cellular Stress Biology, School of Life Sciences, Faculty of Medicine and Life Sciences, Xiamen University, No. 4221, Xiang'an South Road, Xiamen, Fujian 361102, China; State Key Laboratory of Cellular Stress Biology, School of Life Sciences, Faculty of Medicine and Life Sciences, Xiamen University, No. 4221, Xiang'an South Road, Xiamen, Fujian 361102, China; National Institute for Data Science in Health and Medicine, Xiamen University, No. 4221, Xiang'an South Road, Xiamen, Fujian 361102, China

**Keywords:** cellular deconvolution, vision transformer, ATAC-seq, single-cell, chromatin accessibility

## Abstract

The assay for transposase-accessible chromatin with sequencing (ATAC-seq) identifies chromatin accessibility across the genome, crucial for gene expression regulating. However, bulk ATAC-seq obscures cellular heterogeneity, while single-cell ATAC-seq suffers from issues such as sparsity and costliness. To this end, we introduce DECA, a sophisticated deep learning model based on vision transformer to deconvolve cell type information from bulk chromatin accessibility profiles, utilizing single-cell ATAC-seq datasets as reference for enhanced precision and resolution. Notably, patch attention generated by DECA’s multi-head attention mechanism aligns with chromatin interactions detected by Hi-C. Additionally, DECA predicted lineage-specific cell composition changes due to genetic perturbation. The chromatin accessibility signatures predicted by DECA are enriched with cell-type specific genetic variations. Ultimately, we applied DECA on pan-cancer ATAC-seq datasets and demonstrated its capability to deconvolve cell type proportions with clinical significance. Taken together, DECA deconvolves cellular proportions and predicts their chromatin accessibility profiles from bulk chromatin accessibility data, which enable exploring the gene regulatory programs in development and diseases.

## Introduction

Chromatin accessibility is instrumental in controlling gene expression programs during in various biological processes [1]. Assay for transposase-accessible chromatin with high-throughput sequencing (ATAC-seq) is a robust tool for investigating chromatin landscapes [2, 3]. The emergence of single-cell ATAC sequencing (scATAC-seq) techniques provide approaches for assessing chromatin accessibility of individual cells [4, 5]. Several computational methods have been developed for predicting local chromatin interactions using single-cell ATAC-seq data, such as Cicero [6] and JRIM [7]. However, the complexity of library construction, data sparsity, and high costs limit its widespread application. Moreover, current methods like fluorescence-activated cell sorting (FACS) and single-cell multi-omics are also time- and cost-inefficient for real-time monitoring of cell type changes caused by non-coding genome perturbations or drug targeting [8, 9]. Therefore, developing an algorithm to deconvolute cell type information from bulk ATAC data leveraging the existing single-cell ATAC datasets can provide a faster and more cost-effective solution [10, 11].

Deconvolution is complex source separation task [12], where probabilities are assigned to classification labels rather than performing a straightforward multi-class classification. This process is akin to extracting distinct themes from blended text, capturing the proportions of multiple thematic labels alongside their respective descriptions [13]. Recently, most computational deconvolution-related algorithms have faced challenges such as the quality of reference data, generation of ground truth, limitations of computational methodologies, benchmark design and implementation [14]. Existing methods can be broadly categorized into two types: those based on statistical learning or deep learning [15]. Examples of such methods include CIBERSORTx [13], Bisque [16], and DWLS [17], which have been developed based on traditional regression models like non-negative least squares (NNLS) and support vector regression [18]. These tools require pre-selected cell-type-specific features or embedding features to minimize computational overhead while maximizing accuracy. In contrast, TAPE [15] and Cellformer [19] are the deep learning approach that utilizes deep neural networks (DNNs) and transformer-based framework. The encoder layer can learn higher-order latent representations, while the decoder can achieve interpretability within the autoencoder. However, TAPE is designed for processing transcriptomic datasets. In the higher-dimensional ATAC dataset, TAPE might disregard the redundant cis co-accessibility regulatory patterns across different chromosomes, potentially leading to increased noise and overfitting [20]. Additionally, chromatin has a higher-order structure and cis-regulatory elements on accessible regions exhibit cell-type specify functional hierarchy [21, 22], that information would lose in most deep learning models.

In order to address these limitations mentioned above, we proposed a precise and interpretable deep learning framework called DECA (deconvolution of chromatin accessibility profile) to predict cell-type proportion and reconstruct chromatin accessibility. The DECA, by segmenting chromosomes into smaller regions termed patches, utilizes a multi-head attention mechanism to discern interrelations among these patches. This approach effectively reduced the dimensionality of the dataset while capturing patch interactions highly relevant to cell type specificity [23]. Additionally, we implemented an adaptive training framework [15] to optimize prediction outcomes, ensuring the extraction of accurate feature attention weights throughout the training phase.

We constructed simulation and golden benchmark datasets to validate the predictive accuracy and robustness of DECA. The patch-attention weights obtained during the training phase of DECA are equivalent to extracting crucial chromatin co-accessibility patterns from redundant data. Based on the hierarchical structure of chromatin, high-resolution Hi-C data validated the biological interpretability of these filtered attention weights. Moreover, we collected comprehensive cutting-edge research to equip DECA with profound biological insights across diverse scenarios.

## Materials and methods

### Data collection and preprocessing

In this study, we conducted experiments using both public bulk and single-cell ATAC sequencing datasets (Table S1, 2). In this study, we utilized multiple publicly available single-cell ATAC-seq and bulk ATAC-seq datasets. For pseudo-bulk testing, we leveraged single-cell chromatin accessibility profiles from PBMC [24], liver, heart, thymus [25], and pan-cancer [26] datasets, all of which included author-annotated cell type labels. We conducted peak calling on regional and cell type-specific replicates using the ArchR workflow [27] with its MACS implementation. For bulk ATAC-seq data processing, we followed a standard pipeline that included quality control, alignment to the reference genome, and peak calling. Alignment was performed using the Bowtie2 algorithm [28], followed by the identification of chromatin accessibility regions with MACS2 [29], applying appropriate cutoffs to ensure robust peak detection. For accurate comparisons, we only considered shared accessibility regions that were present in both datasets. To facilitate optimal learning from both datasets with differing resolutions, we employed min-max normalization strategy to adjust for discrepancies in sequencing depth and cellular heterogeneity between the bulk and single-cell datasets.

### Simulated datasets benchmarking quantification

To optimize the default parameters of DECA, we generated simulation datasets. We opted for single-cell dataset from bone marrow (BM) samples from healthy donor from Granja *et al.* [24]. This dataset encompasses 12 cell types with precise cell type labels. The widespread utility of the model was demonstrated across multiple tissues as documented by Zhang *et al.* [25], which include thymus, liver and heart samples.

### Golden datasets benchmarking quantification

Additionally, we collected some golden benchmark datasets, wherein samples from the same multi-brain-tissues (caudate, parietal lobe, hippocampus, and substantia nigra), healthy BM and acute myeloid leukemia (AML) samples were characterized with both bulk ATAC and single-cell ATAC sequencing datasets from the same sample. To assess the generalizability of our model, we applied the same parameter combinations that we used with the simulated datasets. This enables for genuine assessment of DECA accuracy.

### Biological insight datasets benchmarking quantification

The feasibility of the model was validated through various aspects, including cell-type differentiation induced by genetic perturbation in hematopoietic lineage [30], enrichment of cell type-specific genetic variation [31, 32] and interpretation of the pan-cancer tumor proportions for clinical stages and prognosis statuses [33].

To predict the differentiation of cell types following the genetic perturbation of transcription factors in hematopoietic lineage, we utilized single-cell ATAC mouse atlas from Cusanovich *et al.* [34]. This atlas covers 13 different organs and tissues, with only BM tissue chosen for pseudo-bulk generation. For predictions, we selected bulk ATAC datasets from hematopoietic lineage after the knockout of regulatory factors [30], which include Smarcd2, Brd9, Rcor1, Chd4 and Rbbp4.

In the section focused on identifying cell-type specific genetic variation, we employed 32 immune cell subtypes obtained through Calderon *et al*. as training references [31]. The library of cell-type specific eQTLs were derived from Ota *et al.* [32] and the enrichment of SNP were calculated by SNPsea [35].

For the interpretation of the pan-cancer tumor clinical classification and prognosis statuses, we employed human cancer single-cell ATAC atlases from Terekhanova *et al.* [26] and human cancer bulk ATAC atlases from Corces *et al.* [33]. Through matching, we obtained six cancer datasets (breast cancer [BRCA], skin cutaneous melanoma [SKCM], endometrioid cancer [UCEC], glioblastoma [GBM], head and neck cancer [HNSCC], and cervical cancer [CESC]) with complete clinical information.

### The DECA framework

**Figure 1:**
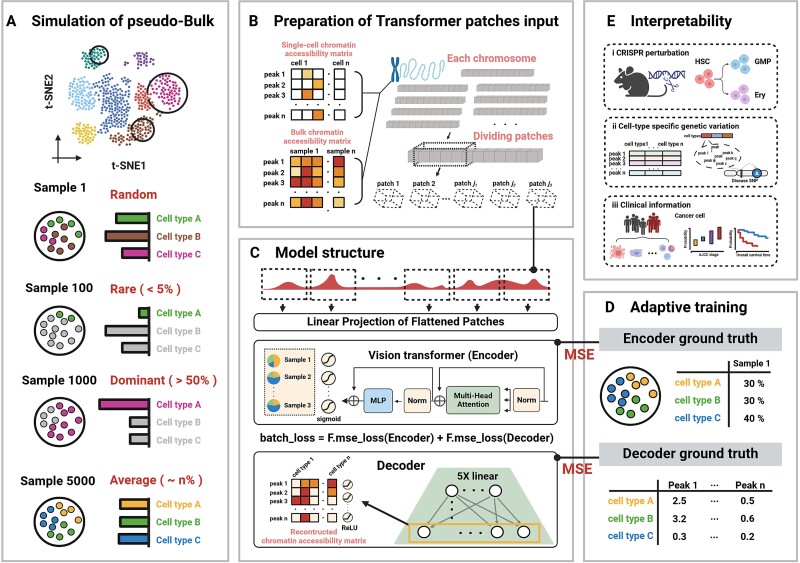
DECA architecture and applications. DECA is a high-resolution deep learning framework for deconvolution from bulk ATAC datasets, predicting cell type proportions and chromatin accessibility matrices. (A) DECA, grounded in single-cell datasets, explores diverse proportion combinations in subpopulations, integrating Random, Rare, Dominant, and Average groups. (B) DECA involves segmenting accessible regions into varying patch sizes. (C) DECA utilizes patches as input for vision transformer (encoder) to capture the proportion information. (D) Supervised training uses generated ground truth for performance evaluation. (E) Biological insights from bulk chromatin accessibility research, including genetic perturbation, genetic variation and clinical application, are illustrated (created with BioRender.com).

As shown in Fig. 1, DECA consists of five modules: simulation of pseudo-bulk samples, preparation of Transformer patches input, model structure, adaptive training, and interpretability.

### Simulation of pseudo-bulk samples

In model construction, generating pseudo-bulk samples from single-cell datasets is essential for training. These pseudo-bulk datasets, derived from single-cell ATAC data with precise cell type labels, are used for training, loss gradient computation, and performance optimization. Following the TAPE method [15], utilizing Dirichlet distribution to simulate the combination of cell type proportions [36]. This approach model diverse biological scenarios, including "Dominant" (when a cell type exceeds 50%), "Rare" (when proportions are below 5%), and "Average" (when proportions are nearly equal).

### Model set-up

#### Splitting accessible regions into patches for embedding

To enhance the Vision Transformer’s processing quality and speed, we leveraged the regulatory relationships between accessible regions. Chromosomes were split into distinct input channels, with each open region converted into corresponding patches for embedding. We designed *j* distinct trainable linear projections (Fig. 1B-C), encompassing both LayerNorm (LN) and Multi-Layer Perceptron (MLP) components [37]. Each patch ${\mathrm{P}}_j$ is transformed, generating an embedding for accessible patches denoted as ${\mathrm{N}}_{\mathrm{j}}\in{\mathrm{R}}^{\mathrm{d}}$, where *d* represents the embedding dimension. Simultaneously, a learnable parameter class token ${\mathrm{P}}_{\mathrm{cls}}\in{\mathrm{R}}^{\mathrm{d}}$ is appended to the patch embedding vectors [37, 38]. The concatenation of these embeddings from *X_0_*, subsequently input into the encoder layer. This approach provides these processes with both the enriched contextual information from the patch embedding and the essential global information carried by the class token. The weight parameters ${\theta}_j$ represent the MLP's linear projection components (Fig. 1).


(1) \begin{equation*} {\mathrm{N}}_{\mathrm{j}}=\mathrm{ML}{\mathrm{P}}_{\mathrm{j}}\left(\mathrm{LN}\left({\mathrm{P}}_{\mathrm{j}}\right);{\mathrm{\theta}}_{\mathrm{j}}\right)\# \end{equation*}

(2) \begin{equation*} {\displaystyle \begin{array}{c}{\mathrm{X}}_0=\left[{\mathrm{P}}_{\mathrm{cls}};{\mathrm{N}}_1;{\mathrm{N}}_2;\dots; {\mathrm{N}}_{\mathrm{j}}\right]\#\end{array}} \end{equation*}

### Vision transformer

To capture intricate features and relationships in accessible region, we used the vision transformer to glean insights from patch embeddings. The vision transformer Encoder comprised $\mathrm{L}$ layers of multi-head self-attention (MSA) and MLP blocks [39]. Layer normalization (LN) was applied before each block, and residual connections were incorporated after each block. The MSA extended self-attention (SA) by concurrently executing k self-attention operations, referred to as "heads", and then projected their concatenated results. For each element in the input $\mathrm{X}\in{\mathrm{R}}^{1+\mathrm{m}\times \mathrm{d}}$, query (q), key (k), and value (v) were computed by linearly projecting the input X with parameters ${\mathrm{W}}_{\mathrm{q},\mathrm{k},\mathrm{v}}\in{\mathrm{R}}^{\mathrm{d}\times 3\frac{\mathrm{d}}{\mathrm{m}}}$.


(3) \begin{equation*} {\displaystyle \begin{array}{c}\mathrm{q},\mathrm{k},\mathrm{v}=\mathrm{X}\cdotp{\mathrm{W}}_{\mathrm{q},\mathrm{k},\mathrm{v}}\#\end{array}} \end{equation*}

(4) \begin{equation*} {\displaystyle \begin{array}{c}\mathrm{SA}\left(\mathrm{X}\right)=\mathrm{v}\cdotp \mathrm{softmax}\left(\frac{\mathrm{q}\cdotp{\mathrm{k}}^{\mathrm{T}}}{\sqrt{\frac{\mathrm{d}}{\mathrm{m}}}}\right)\#\end{array}} \end{equation*}

(5) \begin{equation*} {\displaystyle \begin{array}{c}\mathrm{MSA}\left(\mathrm{X}\right)=\left[\mathrm{S}{\mathrm{A}}_1\left(\mathrm{X}\right);\mathrm{S}{\mathrm{A}}_2\left(\mathrm{X}\right);\dots; \mathrm{S}{\mathrm{A}}_{\mathrm{k}}\left(\mathrm{X}\right)\right]\#\end{array}} \end{equation*}

By inputting the patch embedding ${\mathrm{X}}_0\in{\mathrm{R}}^{1+\mathrm{m}\times \mathrm{d}}$ into the vision transformer encoder, we obtained holistic biological feature representation ${\mathrm{X}}_{\mathrm{l}}$ that was further used to predict cell-type proportions.


(6) \begin{equation*} {\displaystyle \begin{array}{c}{{\mathrm{X}}_{\mathrm{l}}}^{\prime }=\mathrm{MSA}\left(\mathrm{LN}\left({\mathrm{X}}_{\mathrm{l}-1}\right);\mathrm{\alpha} \right)+{\mathrm{X}}_{\mathrm{l}-1},\mathrm{l}=1\dots \mathrm{L}\#\end{array}} \end{equation*}

(7) \begin{equation*} {\displaystyle \begin{array}{c}{\mathrm{X}}_{\mathrm{l}}=\mathrm{MLP}\left(\mathrm{LN}\left({{\mathrm{X}}_{\mathrm{l}}}^{\prime}\right);\mathrm{\beta} \right)+{{\mathrm{X}}_{\mathrm{l}}}^{\prime },\mathrm{l}=1\dots \mathrm{L}\#\end{array}} \end{equation*}

where $\mathrm{\alpha}$ and $\mathrm{\beta}$ were the weight parameters of MSA and MLP in vision transformer encoder (Fig. 1C).

### Cell-type proportions head

Typically, in multi-class classification tasks, the classification head comprises LN and an MLP structure [39]. However, our task was not a straightforward multi-class classification task. In the output process of the MLP, due to the relatively large bias in *softmax*, we chose to substitute it with *sigmoid* instead (Fig. 1C). Classification head was attached to ${\mathrm{X}}_{\mathrm{L}}^0$, representing the class token ${\mathrm{T}}_{\mathrm{cls}}$ learned from the vision transformer encoder, where $\mathrm{\omega}$ was the weight parameters of $\mathrm{MLP}$ in classification head.$${\displaystyle \begin{array}{c}\ \mathrm{p}=\mathrm{sigmoid}\left(\mathrm{MLP}\left(\mathrm{LN}\left({\mathrm{X}}_{\mathrm{L}}^0\right);\mathrm{\omega} \right)\right)\ (8.)\end{array}}$$

### Decoder layer

To enhance DECA's predictive capability for chromatin accessibility in accessible regions (*M*), ${f}_{\gamma }$ is designed as a model without activation layers or biases. It is solely the regularization of the dot product of five weight matrix. Therefore, in this deep learning model, the chromatin accessibility matrix is visible:


(9) \begin{equation*} \mathrm{M}={f}_{\gamma }=\mathrm{ReLU}\left({W}_1\cdotp{W}_2\cdotp{W}_3\cdotp{W}_4\cdotp{W}_5\right) \end{equation*}

The rationale behind formulating such an equation to represent *M*, instead of a singular matrix, is that incorporating more parameters facilitates the model in learning swiftly and effortlessly (Fig. 1).

### Adaptive training process

As previously noted, DECA training occurs in two phases. In the first phase, a large set of pseudo-bulk datasets is used. Each epoch (e) produces two outputs: cell type proportions (P) and the chromatin accessibility matrix (M). To improve accuracy and speed, we adopt a TAPE-inspired adaptive training framework using a greedy iterative optimization approach. Step 1: Using the Mean Squared Error (MSE) loss function to optimize the decoder until the MSE results no longer decrease. Step 2: Using the MSE loss functions (MSE(P, $\tilde{P}$) + MSE(M, $\tilde{M}$)) to optimize the encoder until MSE(P, $\tilde{P}$) no longer decreases (Fig. 1). Here, $\tilde{P}$ and $\tilde{M}$ are the results post-initial training. This method refines encoder and decoder parameters based on the MSE between predictions and ground truth, and between reconstructed and original inputs.


(10) \begin{equation*} MSE\left(P,\tilde{P}\right)=\frac{1}{n}{\sum}_{i=1}^n\kern0.1em {\left({P}_i-{\overset{\hat{\mkern6mu}}{P}}_i\right)}^2 \end{equation*}

(11) \begin{equation*} MSE\left(M,\tilde{M}\right)=\frac{1}{n}{\sum}_{i=1}^n\kern0.1em {\left({M}_i-{\overset{\hat{\mkern6mu}}{M}}_i\right)}^2 \end{equation*}

### Performance evaluation

The linear consistency between the synthetic cell type proportions in pseudo-bulk samples and the predicted proportions of cell types is rigorously evaluated using three metrics: Lin's Concordance Correlation Coefficient (CCC), Mean Absolute Error (MAE), and Spearman's rank Correlation Coefficient (r). The formulas for these metrics are as follows:


(12) \begin{equation*} \mathrm{CCC}=\frac{2\cdotp \operatorname{cov}\left(\mathrm{X},\mathrm{Y}\right)}{\operatorname{var}\left(\mathrm{X}\right)+\operatorname{var}\left(\mathrm{Y}\right)+{\left(\overline{\mathrm{X}}-\overline{\mathrm{Y}}\right)}^2} \end{equation*}

(13) \begin{equation*} \mathrm{MAE}\left(\mathrm{X},\tilde{\mathrm{X}}\right)=\frac{\sum_{\mathrm{i},\mathrm{j}}\kern0.1em \left|{\mathrm{X}}_{\mathrm{i},\mathrm{j}}-{\tilde{\mathrm{X}}}_{\mathrm{i},\mathrm{j}}\right|}{\mathrm{n}\times \mathrm{k}} \end{equation*}

(14) \begin{equation*} \mathrm{r}\left(\mathrm{R}\left(\mathrm{y}\right),\mathrm{R}\left(\hat{\mathrm{y}}\right)\right)=\frac{\mathit{\operatorname{cov}}\left(\mathrm{R}\left(\mathrm{y}\right),\mathrm{R}\left(\hat{\mathrm{y}}\right)\right)}{\sigma_y{\sigma}_{\hat{\mathrm{y}}}} \end{equation*}

For each sample, we compute CCC and MAE to assess the model's specificity and identify overlooked cell types. This helps iteratively adjust training sample distribution, optimize data processing, and refine hyperparameters.

## Results

### DECA overview

DECA is an open-source deep learning framework designed for cell type deconvolution from bulk chromatin accessibility profiles, drawing on the redundant characterization of cell types from scATAC datasets. To handle the challenges of high-dimensional and sparse scATAC datasets and chromatin interactions, DECA integrates vision transformer (ViT) [37] and decoder architectures (Fig. 1). Initially, we created pseudo-bulk samples by randomly combining cells with precise labels, following the Dirichlet distribution (Fig. S1A-D, Fig. S2A-B). These samples reflect various distributions, including Random, Rare, Dominant, and Average (Fig. 1A, see Methods). Next, we extracted consensus chromatin accessible regions shared between single-cell and bulk ATAC datasets, segmenting them by chromosome to focus on interactions within the same chromosome. These regions were then divided into patches for DECA input (Fig. 1B).

Third, DECA employs ViT and decoder architectures, where the encoder mimics initial feature processing by ViT, partitioning features into sub-patches with recorded positional embeddings (Fig. 1C). Cell type proportions are predicted using a MLP head and sigmoid output layer, combined with five linear decoder layers to reconstruct the chromatin accessibility matrix (Fig. 1C). A supervised adaptive training process involves computing MSE for encoder and decoder predictions, optimizing through iterative loss adjustments (Fig. 1C-D, Fig. S2C-D).

Finally, to validate DECA's interpretability, we explored biological insights including: I. Predicting hematopoietic lineage changes due to genetic perturbation; II. Identifying immune cell type-specific genetic variations; III. Inferring tumor cell proportions with clinical relevance from pan-cancer ATAC datasets (Fig. 1E).

### Accuracy and stability evaluation on simulated datasets

**Figure 2:**
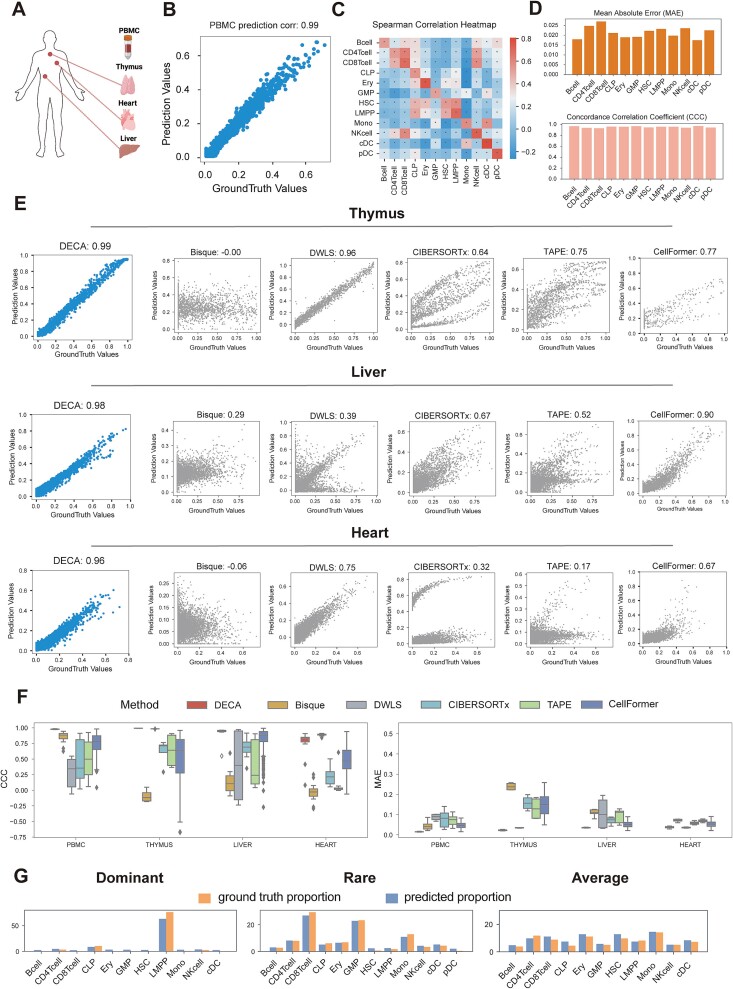
Performance evaluation of DECA on simulation datasets. (A) The schematic diagram depicts the single-cell ATAC datasets from various tissue: PBMC, thymus, liver and heart. (B) The scatter plot represents correlation within PBMC samples. The x-axis represents the cell-type proportion of ground truth, while the y-axis represents the predicted proportion. (C) The heatmap illustrates the spearman correlation between ground truth and reconstructed chromatin accessibility matrices for PBMC samples. P-values were calculated using a two-tailed Spearman rank correlation test. ^*^P < 0.05; ^*^^*^P < 0.01; ^*^^*^^*^P < 0.001; n.s., not significant. (D) Bar plots of DECA accuracy: Lin's concordance correlation coefficient (CCC) and mean absolute error (MAE), where higher CCC and lower MAE indicate better performance (see Methods). (E) These scatter plots of correlations for pseudo-bulk samples using various deconvolution methods (Bisque, DWLS, CIBERSORTx, TAPE, Cellformer) across multi-tissue datasets. (F) These box plots of CCC and MAE for different methods (Bisque, DWLS, CIBERSORTx, TAPE, Cellformer) across multi-tissue datasets. (G) These bar plots of predicted proportions in pseudo-bulk ATAC datasets with different distributions (yellow for actual, blue for predicted).

To evaluate DECA's performance, we simulated pseudo bulk ATAC datasets by merging single cells from peripheral blood mononuclear cells (PBMC), thymus, liver, and heart datasets [24, 25] (Fig. 2A, Table S1, see Methods). This established a ground truth with known cell type proportions and chromatin accessibility. After extensive training, DECA achieved a high spearman correlation coefficient of 0.98 between predicted and actual cell type proportions in the PBMC simulation samples (Fig. 2B, Fig. S3A).

**Figure 3:**
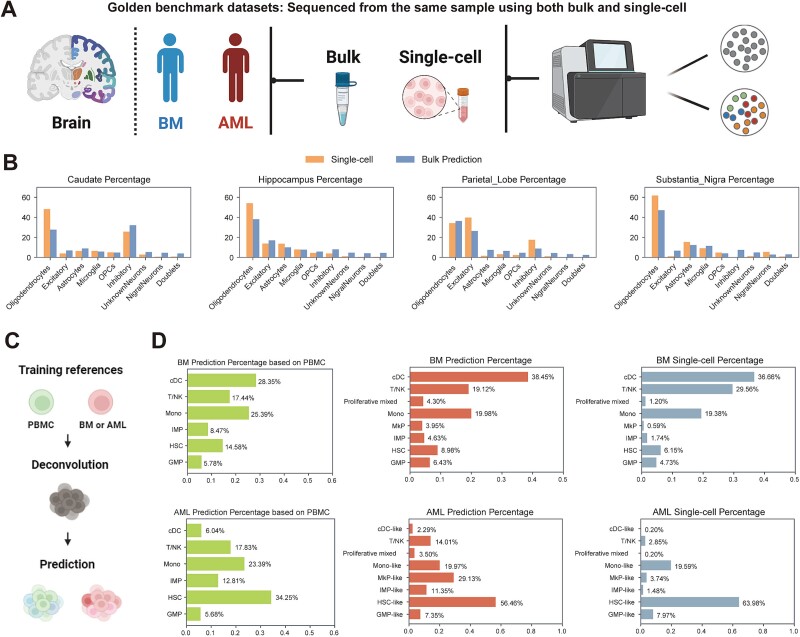
Performance evaluation of DECA on golden benchmark datasets. (A) Schematic of golden benchmark datasets, including single-cell and bulk chromatin accessibility profile from the same donor, covering human brain, healthy bone marrow (BM), and acute myeloid leukemia (AML). (B) These bar plots show predicted (blue) vs. single-cell ground truth (yellow) proportions across four brain regions: caudate, substantia nigra, parietal lobe, and hippocampus. (C) Schematic of DECA predictions for BM and AML samples with different training references. (D) Bar plots of each reference-based predicted (green, red) vs. ground truth (grey) proportions for BM and AML.

DECA also effectively reconstructed cell-type-specific chromatin accessibility profiles. The correlations between reconstructed and true chromatin accessibility matrices for each cell type were significant, with notable similarity in accessibility profiles within lymphoid and myeloid lineages (Fig. 2C, Fig. S3B). The mean absolute error (MAE) and Lin's concordance CCC indicated strong and unbiased performance (Mean (MAE) = 0.023, Mean (CCC) = 0.96) for PBMC dataset predictions (Fig. 2D, Fig. S4A-B). Furthermore, when we select test datasets from different batch studies, the prediction accuracy for the pseudo-bulk model remains consistently robust (Fig. S5A-C).

Additionally, we compared DECA with five deconvolution methods: non-negative least-squares regression (NNLS, Bisque) [16], weighted least squares approach (DWLS) [17], linear regression (CIBERSORTx) [13], deep learning method DNN-based autoencoder (TAPE) [15] and transformer-based separator (Cellformer) [19] (Table S3). Within datasets pertaining to the thymus, liver, and heart [25], DECA outperformed these methods in deconvoluting simulated bulk samples from thymus, liver, and heart datasets, achieving spearman coefficients of 0.99, 0.98, and 0.96, respectively, reflecting strong correlations with synthetic ground truth (Fig. 2E). DECA also demonstrated the best MAE and CCC across various datasets, highlighting its superior performance over existing methods (Fig. 2F). In addition to accuracy and stability, the scalability of the DECA is also crucial for practical applications. Therefore, we evaluated the impact of key hyperparameters within DECA on model performance. We assessed DECA's performance with varying patch sizes (10, 20, 50, 100, 250, 500). The results indicate that patch size influences both training efficiency and performance, but these effects appear to no significant linear trend (Fig. S6A). Therefore, we choose a patch size of 50 for the remaining analysis, balancing prediction accuracy with model interpretability in capturing chromatin accessibility interaction. Additionally, we measured both memory usage and runtime for each tool. Memory usage was comparable across DECA, Cellformer, Bisque, DWLS (Fig. S6B). DECA exhibits moderate runtime, which is longer compared to TAPE, Cellformer, and Bisque, but shorter than DWLS (Fig. S6C).

Considering variations in cell type distributions, DECA was trained on different combinations (Dominant, Rare, Average) (see Methods). Specifically, Lymphoid-primed multipotent progenitors (LMPPs), key in lymphocyte development, were prominent in the "Dominant" state [40] (Fig. 2G). Disease samples like B lymphoma, where B cells are challenging to distinguish, were considered in the "Rare" state [41] (Fig. 2G). And the state of "Average" demonstrates the robustness of DECA in predicting various cell types (Fig. 2G). Taken together, DECA shows strong predictive capability and versatility, making it applicable across various tissues.

### Performance evaluation on golden benchmark datasets

We next test the performance of DECA on the golden benchmark datasets which were comprised of single-cell ATAC and bulk ATAC datasets obtained from the same samples. To this end, we collected datasets from human brain [42], along with healthy BM and AML samples [11] (Table S1, 2, Fig. 3A, see Methods).

The human brain dataset contained four regions of the same donor: the caudate, substantia nigra, parietal lobe, and hippocampus. Using scATAC as reference for training, and perform DECA predictions on bulk ATAC datasets, we observed that DECA swiftly identified "Dominant" and "Rare" cell types within bulk ATAC datasets, such as oligodendrocytes in each region and nigral neuron within caudate region (Fig. 3B, blue). These findings were consistent with reference annotations found in single-cell datasets (Fig. 3B, yellow), providing DECA insights into the dominant cell types involved in the epigenetic regulation of motor control, cognition, attention, and memory at bulk resolution.

To ensure whether DECA is affected by differences in the training dataset, such as batch effects. We utilized publicly available single-cell ATAC datasets from another PBMC sample [24], as well as AML and BM [11], as training references separately (Fig. 3C). The results show that relatively consistent cell type proportions, further validating DECA's robustness to batch effects (Fig. 3D, green, red). We observe significant differences in the distribution of cell proportions between BM and AML, with notable alterations in the proportions of hematopoietic stem cells (HSC) (Fig. 3D, green). Previous studies have revealed parallels between leukemic stem cells and HSCs, particularly those enriched in AML [43]. By using the golden benchmark single-cell dataset as training for predictions, DECA could discern comparable cell type proportion distributions and accessibility profile in the bulk dataset, aligning with the aforementioned results (Fig. 3D, red, grey, Fig. S7–8). These results also revealed DECA's strong capability to eliminate batch effects in diverse training references. Furthermore, the ‘Rare’ state of T/NK cells also be observed (Fig. 3D, red, grey) in BM samples, and the proportions of HSC-like, GMP-like, and Mono-like cells closely mirrored those annotated in the original AML single-cell dataset Fig. 3D, red, grey). Nevertheless, certain inconsistencies surfaced, notably the fluctuating proportion of MkP-like cells in AML samples (Fig. 3D, red, grey). However, there has some variability might be attributed to differences in library construction between single-cell sequencing and bulk sequencing, leading to distortions in the distribution of certain cell types [3].

Overall, these results suggest that DECA perform effectively in golden benchmark datasets, accurately reconstructs the underlying cell-type specific chromatin accessibility and capably eliminate batch effects during the training phase.

### The patch-attention generated by DECA associates with hi-C-detected chromatin interactions

The primary strength of DECA lies in its ability to purify cell-type-specific patch attention by utilizing the comprehensive extraction of chromatin accessibility. In previous studies, researchers have found that chromatin possesses higher-order structure, and cis-regulatory elements located in accessible regions exhibit cell-type-specific functional hierarchies [21, 22]. High-throughput chromosome conformation capture (Hi-C) sequencing technology can detect chromatin interactions [21, 44–46]. We hypothesize that the presence or absence of chromatin interactions is crucial for cell-type specificity and the multi-head attention mechanism in DECA can reflect long-distance chromatin interactions.

**Figure 4:**
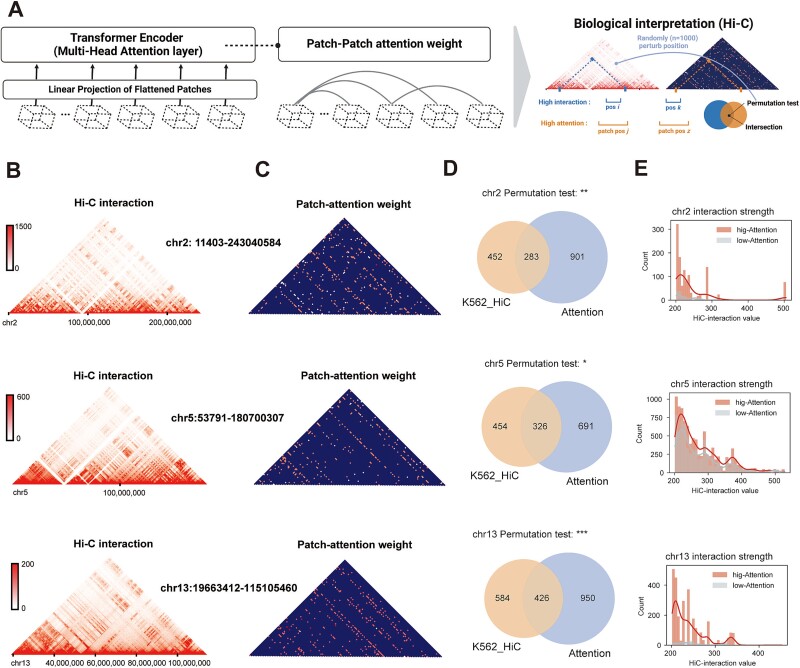
Revealing biological interpretation of the patches-attention generated during DECA training phase. (A) Diagram of attention calculation among patches and its biological interpretation (see Methods). (B) Heatmaps of Hi-C interactions at 20kb resolution across chromosomes. (C) Heatmaps of patch attention weights generated by DECA (see Methods). (D) Venn diagrams showing overlap between genomic locations with high Hi-C interactions and regions with high patch attention weights. The p-values between clinical stages were calculated using permutation testing (50,000 iterations), ^*^P < 0.05; ^*^^*^P < 0.01; ^*^^*^^*^P < 0.001; n.s., not significant. (E) Bar plots of intersecting results between high- and low-attention patches, with line plots using KDE (Kernel Density Estimation) to estimate the probability density function of continuous variables.

Interestingly, we obtained the weights of the attention mechanism between different accessible regions (patches) on the same chromosome during the training process of PBMC datasets [24] (Fig. 4A, see Methods). We compared the attention weights with the Hi-C interactions for each chromosome in K562 (erythroleukemia cells) [47] (Fig. 4B-C). Moreover, compared to the chromatin co-accessibility tools Cicero and JRIM, DECA identified similar interaction patterns, with Hi-C interactions serving as the gold standard for positive validation (Fig. S9A-B). To quantify and validate these similarities, we systematically selected patches characterized by high absolute values of patch-attention weights and Hi-C interaction strength, which predominantly coincided with distinct overlap genome location (Fig. 4A). During the comparison process, we employed permutation test to perturb the genomic positions of Hi-C interactions, and assessed the statistical significance of the overlap calculation (Fig. 4D). This observation suggested that ATAC datasets trained within DECA could partially capture chromatin structure, with the patch-attention weights potentially mirroring interactions among accessible regions. We classified attention weights into High, Median, and Low categories and analyzed their correlation with Hi-C signals across various chromosomal regions. High and Median attention showed significantly stronger Hi-C interactions than Low attention (Fig. S9C). Assessing the magnitude of patch-attention values, we segregated them into two distinct categories and probed the interaction intensity between accessible regions among overlap locations, revealing that these compressed attention weights exhibited heightened genome interaction (Fig. 4E).

Collectively, these results demonstrate that DECA possesses biologically interpretable strengths, where the trained attention weights correlated with chromatin interaction patterns on the same chromosome.

### DECA reveals lineage-specific cell-type composition altered by genetic perturbation

**Figure 5:**
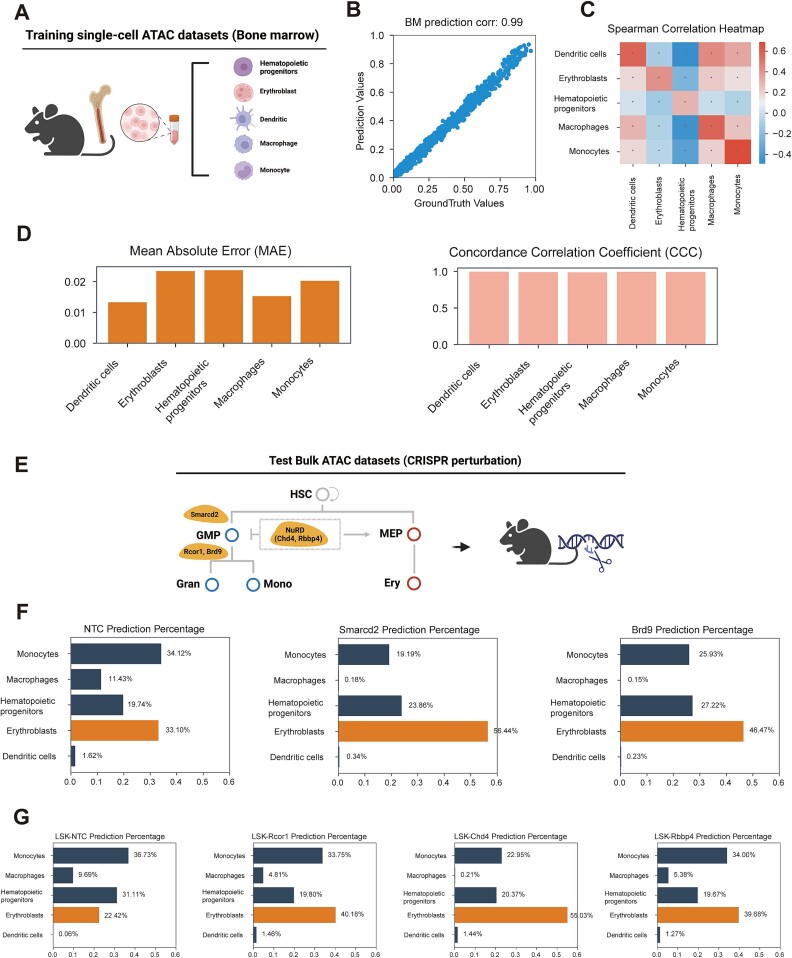
Revealing biological insight of cell-type proportion utilizing perturbation data. (A) Diagram showing public scATAC datasets from mouse bone marrow, including five major cell types. (B) The scatter plot represents correlation within mouse bone marrow samples. The x-axis represents the cell-type proportion of ground truth, while the y-axis represents the predicted proportion. (C) Heatmap validating DECA-generated chromatin accessibility matrices against ground truth using Spearman correlation. P-values were calculated using a two-tailed Spearman rank correlation test. ^*^P < 0.05; ^*^^*^P < 0.01; ^*^^*^^*^P < 0.001; n.s., not significant. (D) Bar plots of DECA prediction accuracy for mouse bone marrow: MAE for prediction accuracy and CCC for consistency with ground truth. (E) Diagram of public bulk ATAC datasets from knockout mice with different transcription factor complexes. (F-G) Bar charts of DECA's predictions on cell-type proportion changes after transcription factor perturbations in hematopoietic lineage (non-treated control (NTC), Smarcd2, Brd9, Rcor1, Chd4, Rbbp4; see Methods).

Lineage differentiation involves extensive chromatin modifications guided by chromatin and transcription factors [48, 49]. Recent studies by Lara-Astiaso et al. identified 142 lineage-specific chromatin factors in mice, highlighting their role in regulating myeloid differentiation [30]. We tested DECA's ability to predict these findings using a single-cell atlas of mouse BM hematopoietic lineages, including hematopoietic progenitors, erythroblasts, dendritic cells, macrophages, and monocytes (Fig. 5A, see Methods). DECA achieved a high correlation of 0.99 between predicted and actual values for pseudo-bulk samples (Fig. 5B). Additionally, DECA effectively traced hematopoietic progenitor lineage to myeloid and erythroid lineages, showing strong correlations (Fig. 5C). And the assessments for each cell type prediction were conducted using MAE and CCC (Mean [MAE] = 0.02, Mean [CCC] = 0.98, Fig. 5D).

In the aforementioned genetic perturbation experiments [30], Lara-Astiaso et al.'s experiments on knockouts of Smarcd2, Brd9, Rcor1, Chd4, and Rbbp4 revealed their importance in hematopoietic stem cell differentiation, confirmed by FACS (Fig. 5E). DECA's predictive analysis of bulk ATAC datasets replicated these findings, showing that Smarcd2 and Brd9 knockouts inhibited myeloid differentiation while promoting erythroid differentiation, compared to the non-treated control (NTC) group (Fig. 5F). Similarly, the knockout of Rcor1, Chd4, and Rbbp4 in vitro cultures of multipotent progenitors (Lin − Sca1 + c-Kit+, LSKs) resulted the sharp accumulation of erythroid (Fig. 5G). This finding is consistent with Lara-Astiaso et al. that suggested the NuRD (Chd4) complex repressive activity proved crucial for terminal erythropoiesis and its depletion induced the accumulation of aberrant erythroid cells [30]. Taken together, these results suggested DECA’s capability to reveal cell-type composition changes due to genetic perturbations in lineage-specific chromatin factors based on bulk chromatin accessibility profiles.

### Chromatin accessibility signatures inferred by DECA is enriched for cell-type-specific genetic variation

Genetic studies have identified numerous variant loci associated with immune-mediated diseases [50]. Understanding the pathogenic mechanisms of these diseases requires insights into the functionality of these genetic variants, particularly under disease-relevant conditions. eQTL and SNP analyses have revealed dynamic genetic variability across various immune conditions and cell types [51], providing a basis to validate DECA’s ability to reconstruct functional chromatin accessibility.

**Figure 6:**
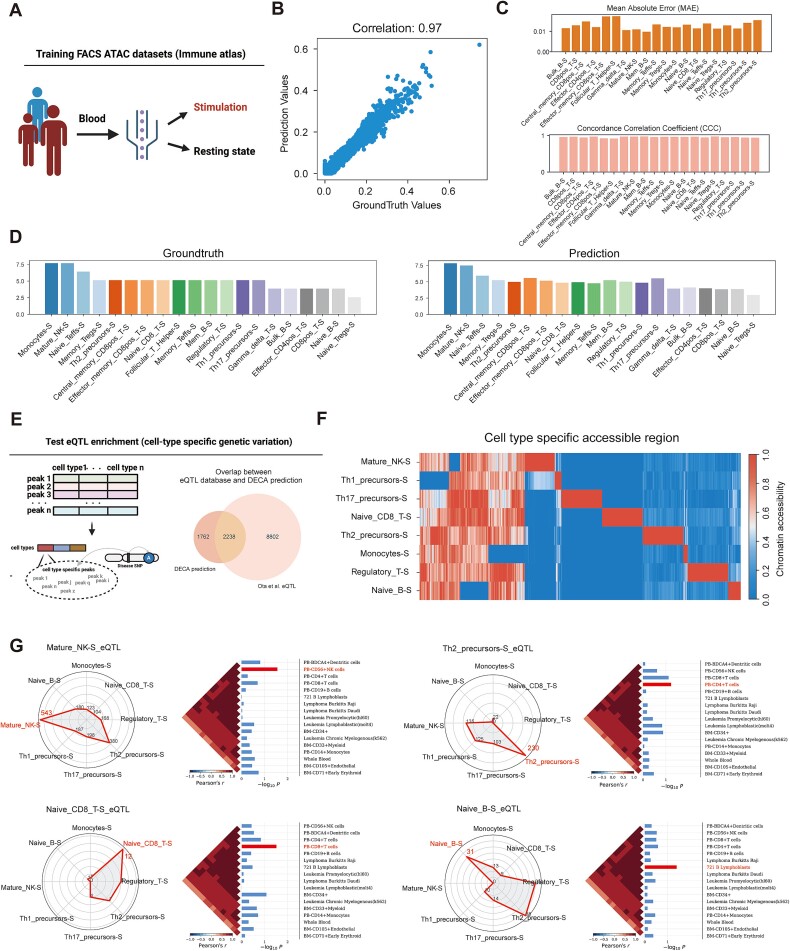
Revealing biological insight of cell-type chromatin accessibility utilizing genetic variation data. (A) Diagram of ATAC datasets from stimulated immune cells in human blood, used for DECA training. (B) The scatter plot represents correlation within immune cell-type atlas samples. The x-axis represents the cell-type proportion of ground truth, while the y-axis represents the predicted proportion. (C) Bar plots of DECA prediction accuracy for the immune atlas: MAE for accuracy and CCC for consistency with ground truth. (D) Bar plots showing similarity between ground truth and predicted cell-type proportions from masked training. (E) Diagram illustrating the use of DECA-generated chromatin accessibility matrices to identify cell-type-specific genetic variations and the overlap between DECA predictions and genetic variations identified by Ota et al. (F) The heatmap shows chromatin accessibility profiles across immune cell types predicted by DECA. Red indicates accessible regions, and blue indicates low accessibility. Cell type-specific peaks highlight chromatin regions unique to each immune cell type. (G) Radar plots showing the enrichment of immune cell-type-specific eQTLs in the specific peaks identified by DECA. Each axis represents an immune cell type, and the plot illustrates the number of overlapping eQTLs between the chromatin accessibility peaks and cell-type-specific eQTLs. These heatmaps and bar plots show cell-type specific SNPs. In the SNPsea analysis, the top cell types with the most significant enrichment on the specific and background peaks identified in the DECA-reconstructed chromatin accessibility profile. The red bars highlight the results with the most significant one-sided P-values. The heatmap displays the Pearson correlation coefficients, ordered by hierarchical clustering using the unweighted pair-group method with arithmetic means (UPGMA).

We used the chromatin accessibility atlas of immune cell types stimulated in vitro, obtained through FACS-based sequencing by Calderon *et al.* [31], as training references (Fig. 6A). DECA achieved a high correlation of 0.97 between predicted and actual values (Fig. 6B). The predictions for each cell type were assessed with MAE and CCC, showing a mean MAE of 0.02 and a mean CCC of 0.95 (Fig. 6C). Intriguingly, even when proportions of twenty cell types were randomly masked during training, DECA accurately predicted cell type proportions from bulk samples (Fig. 6D), indicating its capability to handle diverse cell types and without significant bias.

**Figure 7:**
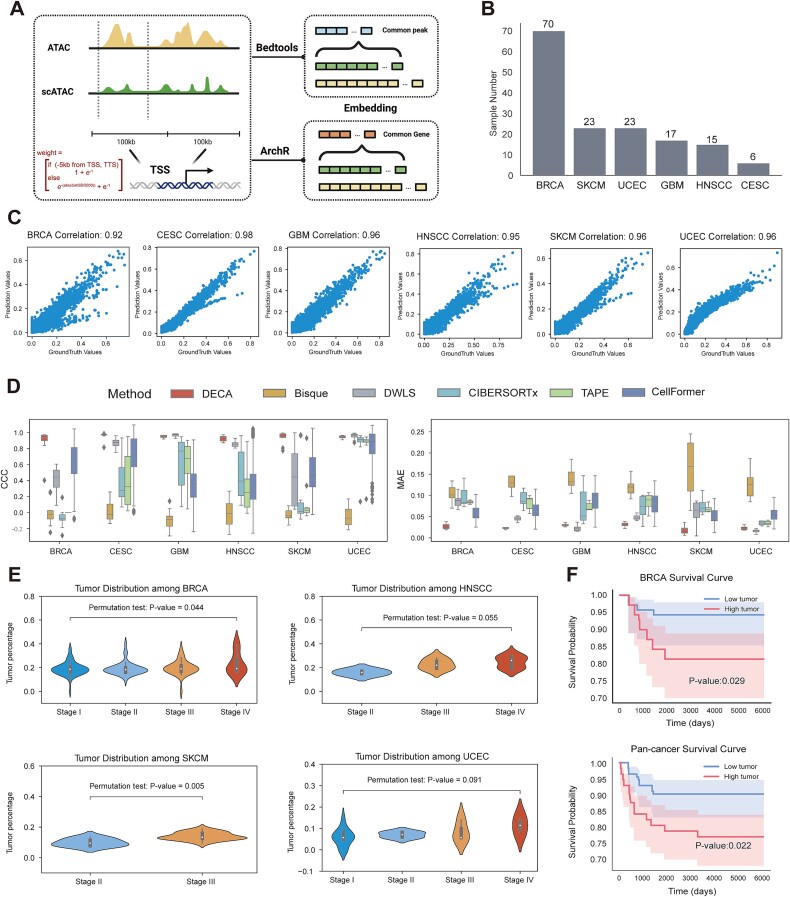
Deconvolution benchmark on pan-cancer datasets with clinical information. (A) Schematic of converting chromatin accessibility matrices into gene activity matrices. (B) Bar plot showing the number of bulk tumors ATAC samples from TCGA. (C) Scatter plot of pseudo-bulk samples, comparing ground truth (x-axis) and predicted proportions (y-axis). (D) Box plots of Lin’s concordance correlation coefficient (CCC) and mean absolute error (MAE) for different deconvolution methods (Bisque, DWLS, CIBERSORTx, TAPE, Cellformer) across pan-cancer datasets (BRCA, CESC, GBM, HNSCC, SKCM, UCEC). (E) Violin plots of predicted tumor cell proportions in samples from patients at different grading stages, colored by different grading strategies. The p-values between clinical stages were calculated using permutation testing (50,000 iterations). (F) Kaplan-Meier survival curves showing the impact of predicted tumor cell proportions on patient outcomes (top: BRCA; bottom: pan-cancer).

We further investigated cell-type-specific chromatin accessibility by analyzing eQTL enrichment identified by Ota *et al.* [32] (Fig. 6E). DECA’s reconstructed chromatin accessibility matrix was used to identify accessible regions specific to certain cell types, revealing significant overlaps with eQTLs and differentially accessible regions (Fig. 6F). SNPsea analysis also showed significant enrichment of cell-type-specific SNPs within the peaks extracted by DECA across all examined cell types [35] (Fig. 6G). Therefore, DECA effectively reconstructs cell-type-specific chromatin accessibility and links these features to disease phenotypes in bulk ATAC datasets.

### Deconvolution on pan-cancer ATAC-seq datasets provides clinical significance

ATAC-seq datasets can be transformed into three distinct data dimensions: continuous bins, peaks, and genes. The vast feature embedding of bins, which spans the entire genome, results in billions of features and significant computational challenges [52]. Peaks, being non-predefined and variable across datasets, complicate the direct application of pre-trained models. Therefore, uniformity in peak calling and invocation processes is essential (see Methods). In this section, DECA also utilized gene activity scores from human cancer single-cell ATAC atlases from Terekhanova et al. [26] as training references, and prediction for human cancer bulk ATAC atlases from Corces et al. [33]. The datasets included pan-cancer ATAC-seq data from various cancers such as BRCA, CESC, GBM, HNSCC, SKCM, and UCEC (Fig. 7A-B). This allowed DECA to operate within smaller feature embeddings. In the deconvolution of pseudo-bulk samples [33], DECA achieved correlations of 0.92, 0.98, 0.96, 0.95, 0.96, and 0.96 between predicted and actual values (Fig. 7C, Fig. S10A-B).

Comparative analysis showed DECA outperformed Bisque, DWLS, CIBERSORTx, and TAPE, with MAE of 0.023 and CCC of 0.96 (Fig. 7D). DECA also identified cell-type-specific gene activity and pathway enrichments that aligned well with known markers (Table S4). We opted for permutation testing to assess the statistical significance between tumor proportions and clinical stages. The results indicate that BRCA and SKCM are statistically significant, while non-significant findings may be influenced by sample size and missing stage information (Fig. 7E). Moreover, DECA-predicted higher tumor proportions are linked to worse prognosis and shorter survival (Fig. 7F). Notably, DECA's predicted tumor proportions correlated with clinical stages and survival status from TCGA samples, reflecting its clinical relevance [53–56]. These findings underscore DECA’s role in identifying clinically significant cell types from ATAC datasets.

## Discussion

The bulk ATAC sequencing technique [2, 3] is extensively used for exploring chromatin accessible regions in various biological contexts related to epigenetic regulation. Single-cell ATAC-seq, while effective for detecting chromatin accessibility in specific cell types, can be costly and may result in significant missing events in the open chromatin matrix, potentially obscuring positive results [4, 5]. Currently, methods for deconvolving accessible sequencing datasets from bulk tissue to obtain cell-type specific states and chromatin accessibility information are limited [19, 57, 58].

To address this, we developed a deep learning-based model called DECA, specifically designed for ATAC datasets. DECA segments chromosomes into equally sized patches, which are then processed using a ViT [37]. The multi-head attention mechanism of ViT enables DECA to capture long-range dependencies within sequences, facilitating a comprehensive understanding of co-accessibility patterns in the genome and effectively extracting relevant features from the patches [39]. DECA's key innovations include:

1. Utilizing ViT's multi-head attention to segment chromosomes into different patches, capturing attention weights between patches to better identify consensus features of accessible regions for cell-type identification.
2. Offering an adaptive open-source training framework for deconvolving multiple tissues, determining cell type proportions and chromatin accessibility within bulk samples to reveal genomic patterns.

DECA also shows strong biological interpretability. The patch-attention weights obtained during training exhibit topologically associated domain boundaries similar to Hi-C [21, 44–46], highlighting ViT's advantage in capturing long-range dependencies. However, patch-attention does not effectively capture the interactions between adjacent patches as accuracy as Hi-C. This might be related to factors such as DECA’s sparse attention mechanisms or hard selection strategies, and the absence of Hi-C data during the training phase. DECA's performance was validated using brain, BM, and acute myeloid leukemia (AML) samples, where it demonstrated superior results on benchmark datasets and effectively predicted cell type proportions and functional chromatin accessibility [30–32, 34]. However, we also observed moderate divergences between prediction and real cell type proportion for certain cell types in specific brain regions, such as inhibitory neurons in the substantia nigra. Although we excluded this is due to the specific cell-type biases during the DECA training phase (Fig. S11), it requires further exploration and validation in the future. Finally, deconvolution of gene activity matrix obtained from pan-cancer ATAC datasets revealed the significant superiority of DECA in low-dimensional [26, 33].

Despite its strengths, DECA relies on reference ATAC datasets from labeled single-cell or FACS-selected data, necessitating careful data preprocessing. In conclusion, DECA provides a cost-effective, efficient method for analyzing chromatin accessibility related to cell specificity and disease progression, offering valuable insights for epigenetic research.

**Key Points**

- DECA, a ViT-based deep learning model, to capture consensus features of cell-type-specific accessible regions using multi-head attention.
- DECA learns TAD-like boundary structures during training and effectively reflects changes in cell composition due to genetic perturbations, cell-type variation, and pan-cancer clinical relevance.
- DECA outperformed existing deconvolution methods in performance, robustness and strong biological interpretability on profound biological insights across diverse scenarios.

## Supplementary Material

supp1_bbaf069

supp2_bbaf069

supp3_bbaf069

supp4_bbaf069

supp5_bbaf069

supp6_bbaf069

supp7_bbaf069

supp8_bbaf069

supp9_bbaf069

supp10_bbaf069

supp11_bbaf069

Table_S1_bbaf069

Table_S2_bbaf069

Table_S3_bbaf069

Table_S4_bbaf069

Supplementary_Fig_legend_Highlight_0107_bbaf069

## Data Availability

All datasets analyzed were previously published (Table S1, 2). For simulation datasets, we used single-cell ATAC-seq from PBMCs of healthy donors (https://github.com/GreenleafLab/MPAL-Single-Cell-2019) and multiple tissue datasets from Ren et al. (https://www.encodeproject.org/entexmatrix/?type=Experiment&status=released&internal_tags=ENTEx). Benchmark datasets included bulk and single-cell ATAC-seq from the same samples, such as human brain data (GSE147672) and healthy BM and AML samples from a previous study [11]. For biological insights, we used single-cell ATAC-seq from hematopoietic lineage (GSE213506) and bulk ATAC-seq from BM (GSM3034623), as well as immune cell-type data (GSE129785) and pan-cancer datasets from https://data.humantumoratlas.org/ and The Cancer Genome Atlas (TCGA).

## Benchmark comparison and settings for existing methods

To evaluate the performance of DECA compared to the existing methods, we selected several representative approaches for comparison. Benchmark tests for deconvolution performance on simulation and golden benchmark datasets were conducted using CIBERSORTx, DWLS, TAPE, and Bisque.

(i) Bisque (v 1.0.5, https://github.com/cozygene/bisque, data pre-processing is done by Biobase (R package), and feature extraction uses the function *Reference Based Decomposition*.
(ii) CIBERSORTx (https://cibersortx.stanford.edu/index.php)
(iii) DWLS (https://github.com/dtsoucas/DWLS)
(iv) TAPE (v1.1.2, https://github.com/poseidonchan/TAPE, data pre-processing is carried out using Pandas).

The mentioned methods were tested following the guidelines and tutorials provided by their respective software packages (Table S3).

## Data and code availability

For future research, the open-source implementation of DECA is available at https://github.com/xmuhuanglab/DECA.

## References

[1] Klemm SL, Shipony Z, Greenleaf WJ. Chromatin accessibility and the regulatory epigenome. Nat Rev Genet 2019;20:207–20. 10.1038/s41576-018-0089-8. 30675018

[2] Buenrostro JD, Giresi PG, Zaba LC. et al. Transposition of native chromatin for fast and sensitive epigenomic profiling of open chromatin, DNA-binding proteins and nucleosome position. Nat Methods 2013;10:1213–8. 10.1038/nmeth.2688. 24097267 PMC3959825

[3] Grandi FC, Modi H, Kampman L. et al. Chromatin accessibility profiling by ATAC-seq. Nat Protoc 2022;17:1518–52. 10.1038/s41596-022-00692-9. 35478247 PMC9189070

[4] Satpathy AT, Granja JM, Yost KE. et al. Massively parallel single-cell chromatin landscapes of human immune cell development and intratumoral T cell exhaustion. Nat Biotechnol 2019;37:925–36. 10.1038/s41587-019-0206-z. 31375813 PMC7299161

[5] Baek S, Lee I. Single-cell ATAC sequencing analysis: from data preprocessing to hypothesis generation. Comput Struct Biotechnol J 2020;18:1429–39. 10.1016/j.csbj.2020.06.012. 32637041 PMC7327298

[6] Pliner HA, Packer JS, McFaline-Figueroa JL. et al. Cicero predicts cis-regulatory DNA interactions from single-cell chromatin accessibility data. Mol Cell 2018;71:858–871.e8. 10.1016/j.molcel.2018.06.044. 30078726 PMC6582963

[7] Dong K, Zhang S. Joint reconstruction of cis-regulatory interaction networks across multiple tissues using single-cell chromatin accessibility data. Brief Bioinform 2021;22:22. 10.1093/bib/bbaa120. PMC8138825 32578841

[8] Chen H, Lareau C, Andreani T. et al. Assessment of computational methods for the analysis of single-cell ATAC-seq data. Genome Biol 2019;20:241. 10.1186/s13059-019-1854-5. 31739806 PMC6859644

[9] Bravo González-Blas C, Minnoye L, Papasokrati D. et al. cisTopic: Cis-regulatory topic modeling on single-cell ATAC-seq data. Nat Methods 2019;16:397–400. 10.1038/s41592-019-0367-1. 30962623 PMC6517279

[10] Avila Cobos F, Alquicira-Hernandez J, Powell JE. et al. Benchmarking of cell type deconvolution pipelines for transcriptomics data. Nat Commun 2020;11:5650. 10.1038/s41467-020-19015-1. 33159064 PMC7648640

[11] Corces MR, Buenrostro JD, Wu B. et al. Lineage-specific and single-cell chromatin accessibility charts human hematopoiesis and leukemia evolution. Nat Genet 2016;48:1193–203. 10.1038/ng.3646. 27526324 PMC5042844

[12] Roweis S . One microphone source separation. Advances in neural information processing systems 2000;13:763–69.

[13] Newman AM, Steen CB, Liu CL. et al. Determining cell type abundance and expression from bulk tissues with digital cytometry. Nat Biotechnol 2019;37:773–82. 10.1038/s41587-019-0114-2. 31061481 PMC6610714

[14] Garmire LX, Li Y, Huang Q. et al. Challenges and perspectives in computational deconvolution of genomics data. Nat Methods 2024;21:391–400. 10.1038/s41592-023-02166-6. 38374264

[15] Chen Y, Wang Y, Chen Y. et al. Deep autoencoder for interpretable tissue-adaptive deconvolution and cell-type-specific gene analysis. Nat Commun 2022;13:6735. 10.1038/s41467-022-34550-9. 36347853 PMC9641692

[16] Jew B, Alvarez M, Rahmani E. et al. Accurate estimation of cell composition in bulk expression through robust integration of single-cell information. Nat Commun 2020;11:1971. 10.1038/s41467-020-15816-6. 32332754 PMC7181686

[17] Tsoucas D, Dong R, Chen H. et al. Accurate estimation of cell-type composition from gene expression data. Nat Commun 2019;10:2975. 10.1038/s41467-019-10802-z. 31278265 PMC6611906

[18] Tran KA, Addala V, Johnston RL. et al. Performance of tumour microenvironment deconvolution methods in breast cancer using single-cell simulated bulk mixtures. Nat Commun 2023;14:5758. 10.1038/s41467-023-41385-5. 37717006 PMC10505141

[19] Berson E, Sreenivas A, Phongpreecha T. et al. Whole genome deconvolution unveils Alzheimer's resilient epigenetic signature. Nat Commun 2023;14:4947. 10.1038/s41467-023-40611-4. 37587197 PMC10432546

[20] Gong W, Dsouza N, Garry DJ. SeATAC: a tool for exploring the chromatin landscape and the role of pioneer factors. Genome Biol 2023;24:125. 10.1186/s13059-023-02954-5. 37218013 PMC10204251

[21] Huang J, Li K, Cai W. et al. Dissecting super-enhancer hierarchy based on chromatin interactions. Nat Commun 2018;9:943. 10.1038/s41467-018-03279-9. 29507293 PMC5838163

[22] Hnisz D, Abraham BJ, Lee TI. et al. Super-enhancers in the control of cell identity and disease. Cell 2013;155:934–47. 10.1016/j.cell.2013.09.053. 24119843 PMC3841062

[23] Alharthi AG, Alzahrani SM. Do it the transformer way: a comprehensive review of brain and vision transformers for autism spectrum disorder diagnosis and classification. Comput Biol Med 2023;167:107667. 10.1016/j.compbiomed.2023.107667. 37939407

[24] Granja JM, Klemm S, McGinnis LM. et al. Single-cell multiomic analysis identifies regulatory programs in mixed-phenotype acute leukemia. Nat Biotechnol 2019;37:1458–65. 10.1038/s41587-019-0332-7. 31792411 PMC7258684

[25] Zhang K, Hocker JD, Miller M. et al. A single-cell atlas of chromatin accessibility in the human genome. Cell 2021;184:5985–6001.e19. 10.1016/j.cell.2021.10.024. 34774128 PMC8664161

[26] Terekhanova NV, Karpova A, Liang WW. et al. Epigenetic regulation during cancer transitions across 11 tumour types. Nature 2023;623:432–41. 10.1038/s41586-023-06682-5. 37914932 PMC10632147

[27] Granja JM, Corces MR, Pierce SE. et al. ArchR is a scalable software package for integrative single-cell chromatin accessibility analysis. Nat Genet 2021;53:403–11. 10.1038/s41588-021-00790-6. 33633365 PMC8012210

[28] Langmead B, Salzberg SL. Fast gapped-read alignment with bowtie 2. Nat Methods 2012;9:357–9. 10.1038/nmeth.1923. 22388286 PMC3322381

[29] Zhang Y, Liu T, Meyer CA. et al. Model-based analysis of ChIP-Seq (MACS). Genome Biol 2008;9:R137. 10.1186/gb-2008-9-9-r137. 18798982 PMC2592715

[30] Lara-Astiaso D, Goñi-Salaverri A, Mendieta-Esteban J. et al. In vivo screening characterizes chromatin factor functions during normal and malignant hematopoiesis. Nat Genet 2023;55:1542–54. 10.1038/s41588-023-01471-2. 37580596 PMC10484791

[31] Calderon D, Nguyen MLT, Mezger A. et al. Landscape of stimulation-responsive chromatin across diverse human immune cells. Nat Genet 2019;51:1494–505. 10.1038/s41588-019-0505-9. 31570894 PMC6858557

[32] Ota M, Nagafuchi Y, Hatano H. et al. Dynamic landscape of immune cell-specific gene regulation in immune-mediated diseases. Cell 2021;184:3006–3021.e17. 10.1016/j.cell.2021.03.056. 33930287

[33] Corces MR, Granja JM, Shams S. et al. The chromatin accessibility landscape of primary human cancers. Science 2018;362. 10.1126/science.aav1898 PMC6408149 30361341

[34] Cusanovich DA, Hill AJ, Aghamirzaie D. et al. A single-cell atlas of In vivo mammalian chromatin accessibility. Cell 2018;174:1309–1324.e18. 10.1016/j.cell.2018.06.052. 30078704 PMC6158300

[35] Slowikowski K, Hu X, Raychaudhuri S. SNPsea: an algorithm to identify cell types, tissues and pathways affected by risk loci. Bioinformatics 2014;30:2496–7. 10.1093/bioinformatics/btu326. 24813542 PMC4147889

[36] Harris CR, Millman KJ, van der Walt SJ. et al. Array programming with NumPy. Nature 2020;585:357–62. 10.1038/s41586-020-2649-2. 32939066 PMC7759461

[37] Dosovitskiy A, Beyer L, Kolesnikov A. et al. An image is worth 16x16 words: transformers for image recognition at scale. ArXiv 2020; abs/2010.11929.

[38] Xie J, Chen Y, Luo S. et al. Tracing unknown tumor origins with a biological-pathway-based transformer model. Cell Rep Methods 2024;4:100797. 10.1016/j.crmeth.2024.100797. 38889685 PMC11228371

[39] Ranftl R, Bochkovskiy A, Koltun V. Vision transformers for dense prediction. In: Proceedings of the IEEE/CVF International Conference on Computer Vision 2021, 12159–68.

[40] Cumano A, Berthault C, Ramond C. et al. New molecular insights into immune cell development. Annu Rev Immunol 2019;37:497–519. 10.1146/annurev-immunol-042718-041319. 31026413

[41] Wang X, Duan M, Li J. et al. MarsGT: multi-omics analysis for rare population inference using single-cell graph transformer. Nat Commun 2024;15:338. 10.1038/s41467-023-44570-8. 38184630 PMC10771517

[42] Corces MR, Shcherbina A, Kundu S. et al. Single-cell epigenomic analyses implicate candidate causal variants at inherited risk loci for Alzheimer's and Parkinson's diseases. Nat Genet 2020;52:1158–68. 10.1038/s41588-020-00721-x. 33106633 PMC7606627

[43] Gal H, Amariglio N, Trakhtenbrot L. et al. Gene expression profiles of AML derived stem cells; similarity to hematopoietic stem cells. Leukemia 2006;20:2147–54. 10.1038/sj.leu.2404401. 17039238

[44] Dixon JR, Selvaraj S, Yue F. et al. Topological domains in mammalian genomes identified by analysis of chromatin interactions. Nature 2012;485:376–80. 10.1038/nature11082. 22495300 PMC3356448

[45] Hou C, Li L, Qin ZS. et al. Gene density, transcription, and insulators contribute to the partition of the drosophila genome into physical domains. Mol Cell 2012;48:471–84. 10.1016/j.molcel.2012.08.031. 23041285 PMC3496039

[46] Sexton T, Yaffe E, Kenigsberg E. et al. Three-dimensional folding and functional organization principles of the drosophila genome. Cell 2012;148:458–72. 10.1016/j.cell.2012.01.010. 22265598

[47] Rao SS, Huntley MH, Durand NC. et al. A 3D map of the human genome at kilobase resolution reveals principles of chromatin looping. Cell 2014;159:1665–80. 10.1016/j.cell.2014.11.021. 25497547 PMC5635824

[48] Ding Y, Liu Z, Liu F. Transcriptional and epigenetic control of hematopoietic stem cell fate decisions in vertebrates. Dev Biol 2021;475:156–64. 10.1016/j.ydbio.2021.03.003. 33689804

[49] Al-Mousawi J, Boskovic A. Transcriptional and epigenetic control of early life cell fate decisions. Curr Opin Oncol 2022;34:148–54. 10.1097/CCO.0000000000000814. 35025815

[50] Zhang J, Zhao H. eQTL studies: from bulk tissues to single cells. J Genet Genomics 2023;50:925–33. 10.1016/j.jgg.2023.05.003. 37207929 PMC10656365

[51] Yazar S, Alquicira-Hernandez J, Wing K. et al. Single-cell eQTL mapping identifies cell type-specific genetic control of autoimmune disease. Science 2022;376:eabf3041. 10.1126/science.abf3041. 35389779

[52] Ma W, Lu J, Wu H. Cellcano: Supervised cell type identification for single cell ATAC-seq data. Nat Commun 2023;14:1864. 10.1038/s41467-023-37439-3. 37012226 PMC10070275

[53] Schanne DH, Koch A, Elicin O. et al. Prognostic and predictive biomarkers in head and neck squamous cell carcinoma treated with radiotherapy-a. Systematic Review, Biomedicines 2022;10:10. 10.3390/biomedicines10123288. PMC9775486 36552043

[54] Haemmerle M, Stone RL, Menter DG. et al. The platelet lifeline to cancer: challenges and opportunities. Cancer Cell 2018;33:965–83. 10.1016/j.ccell.2018.03.002. 29657130 PMC5997503

[55] Stone RL, Nick AM, McNeish IA. et al. Paraneoplastic thrombocytosis in ovarian cancer. N Engl J Med 2012;366:610–8. 10.1056/NEJMoa1110352. 22335738 PMC3296780

[56] Tong M, Luo S, Gu L. et al. SIMarker: cellular similarity detection and its application to diagnosis and prognosis of liver cancer. Comput Biol Med 2024;171:108113. 10.1016/j.compbiomed.2024.108113. 38368754

[57] Newman AM, Liu CL, Green MR. et al. Robust enumeration of cell subsets from tissue expression profiles. Nat Methods 2015;12:453–7. 10.1038/nmeth.3337. 25822800 PMC4739640

[58] Avila Cobos F, Vandesompele J, Mestdagh P. et al. Computational deconvolution of transcriptomics data from mixed cell populations. Bioinformatics 2018;34:1969–79. 10.1093/bioinformatics/bty019. 29351586

