# Supplementary figures and images for: DECA: harnessing interpretable transformer model for cellular deconvolution of chromatin accessibility profile
Source: Brief Bioinform. 2025 Feb 23;26(1):bbaf069. doi: 10.1093/bib/bbaf069 (PMC11847511; doi:10.1093/bib/bbaf069)

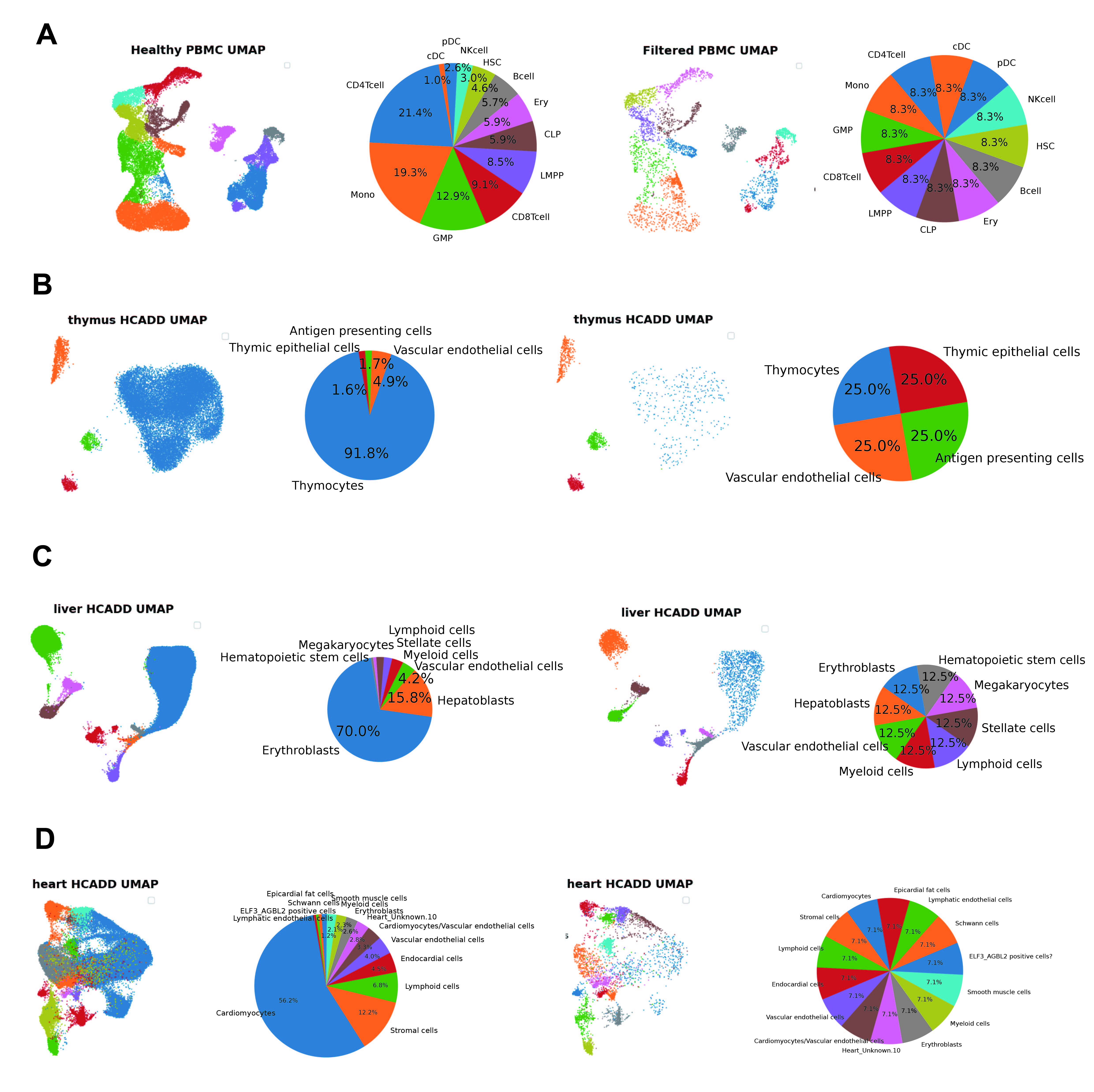

Supplement: supp1_bbaf069 [file supp1_bbaf069.jpeg]

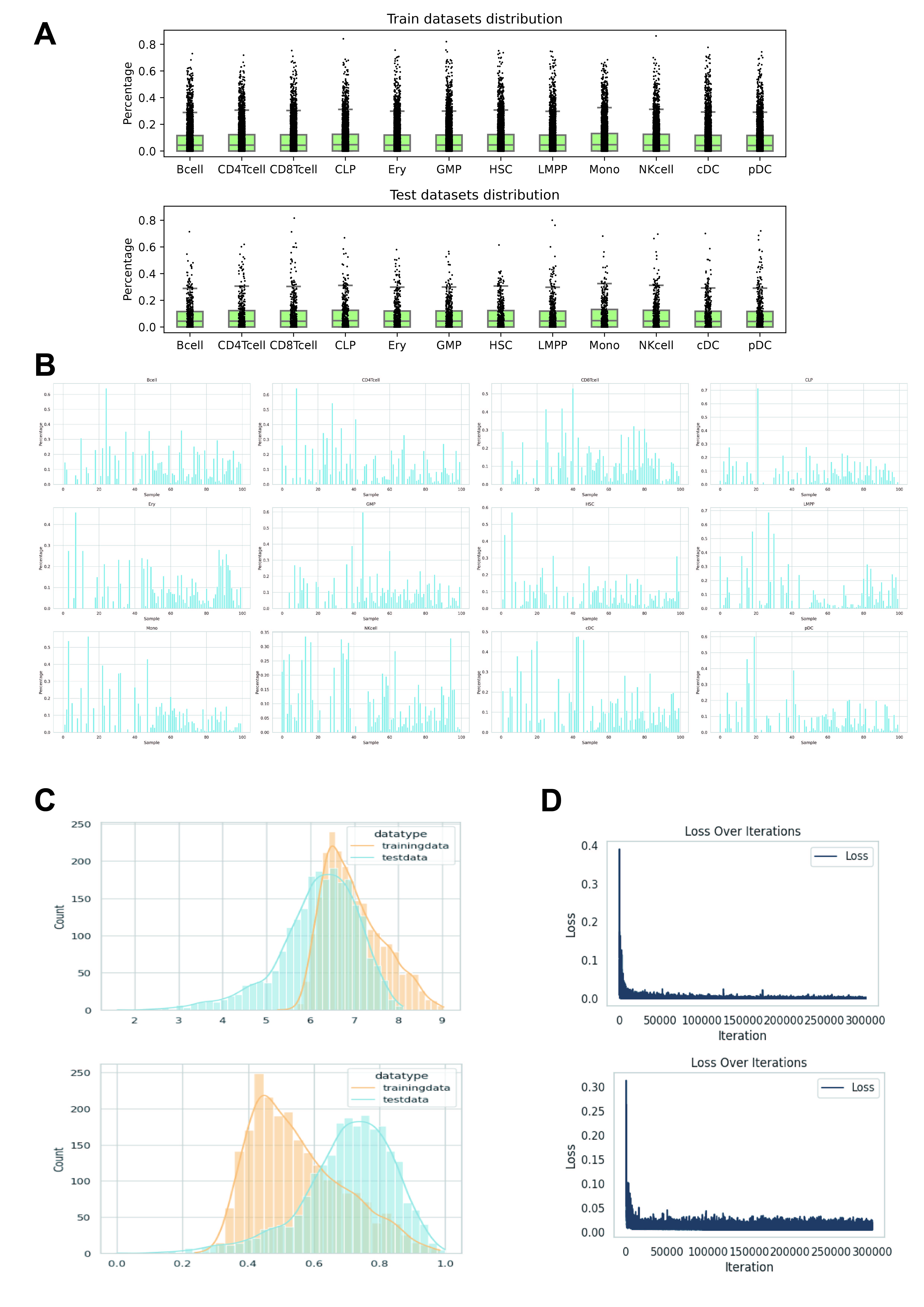

Supplement: supp2_bbaf069 [file supp2_bbaf069.jpeg]

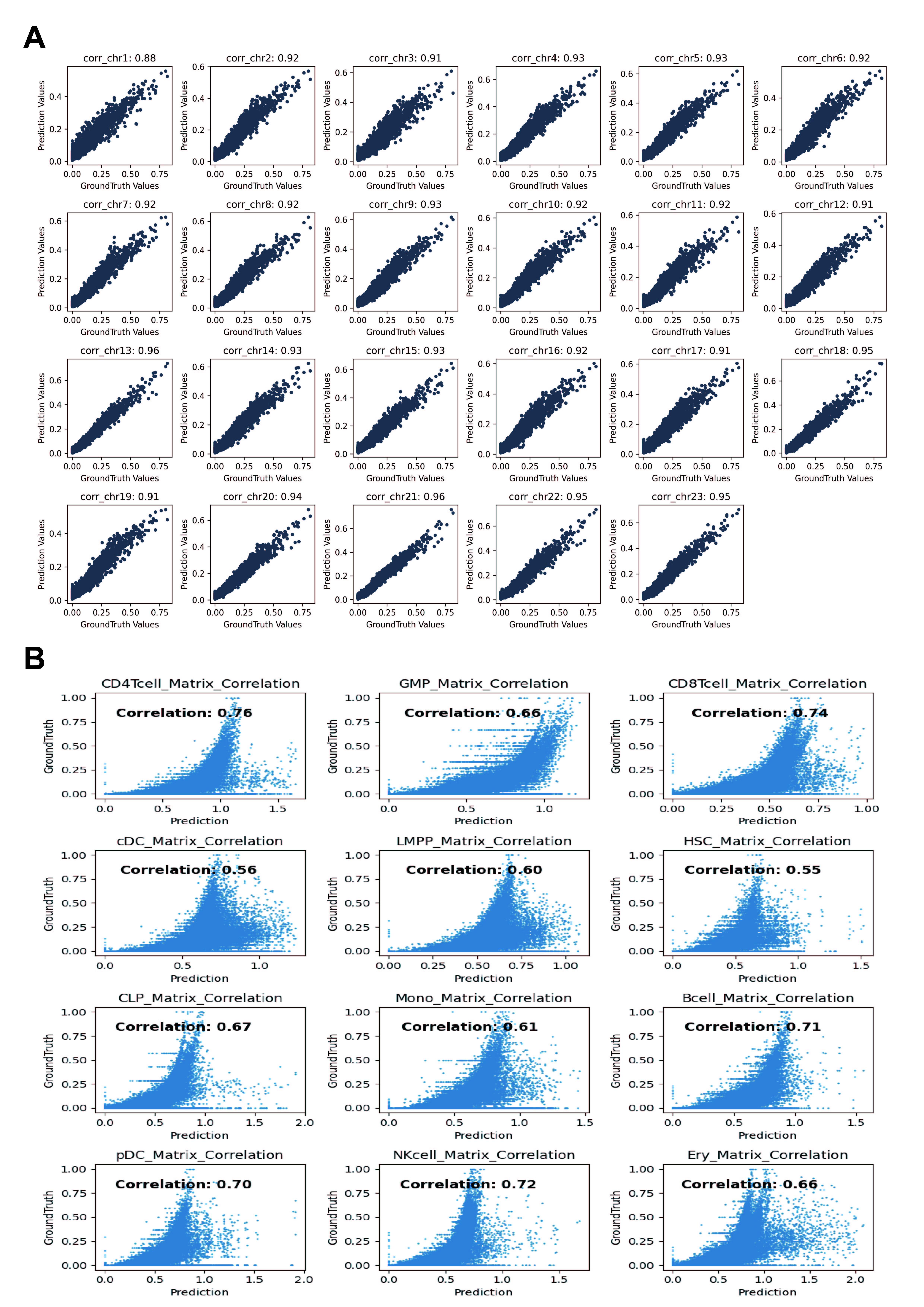

Supplement: supp3_bbaf069 [file supp3_bbaf069.jpeg]

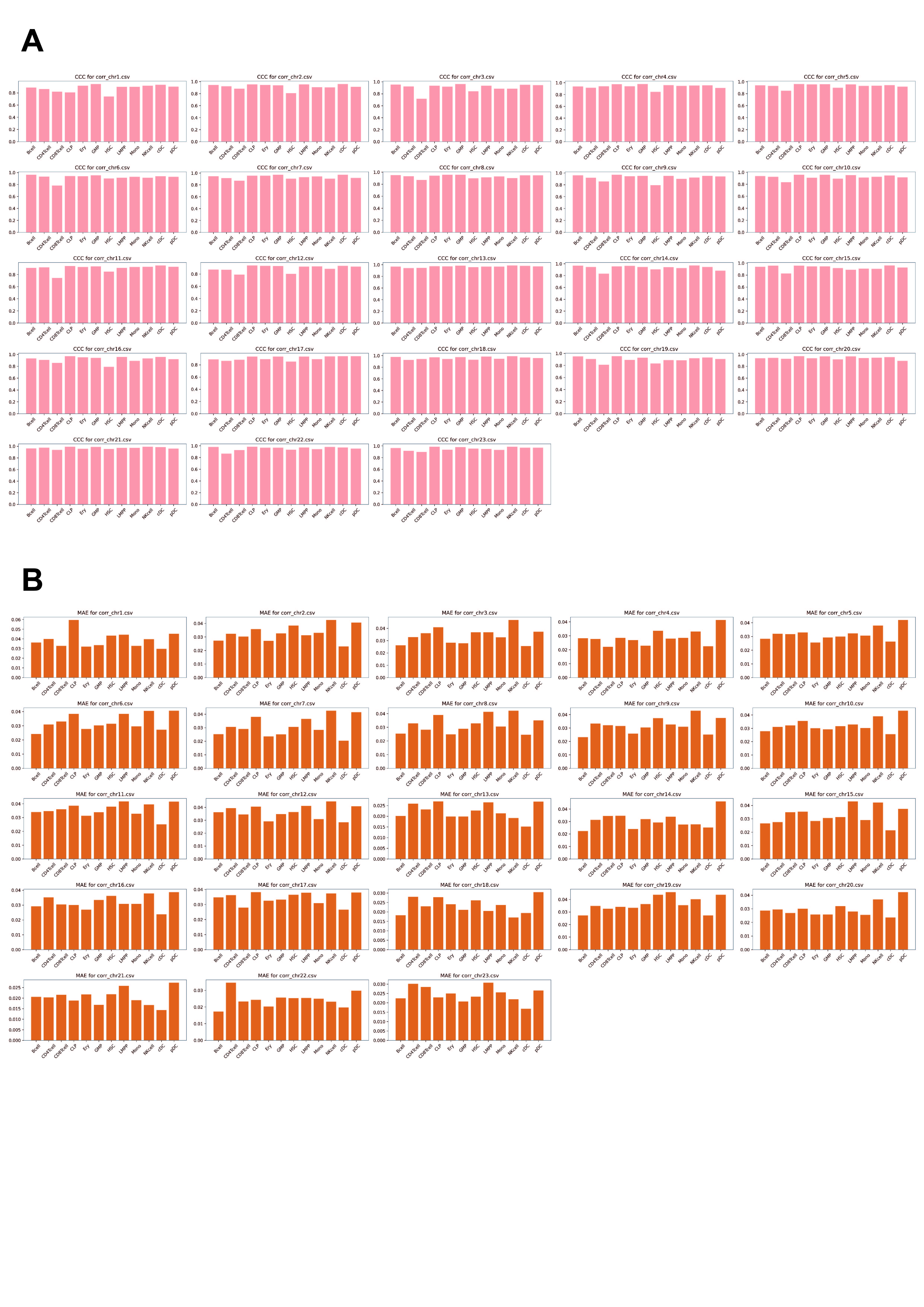

Supplement: supp4_bbaf069 [file supp4_bbaf069.jpeg]

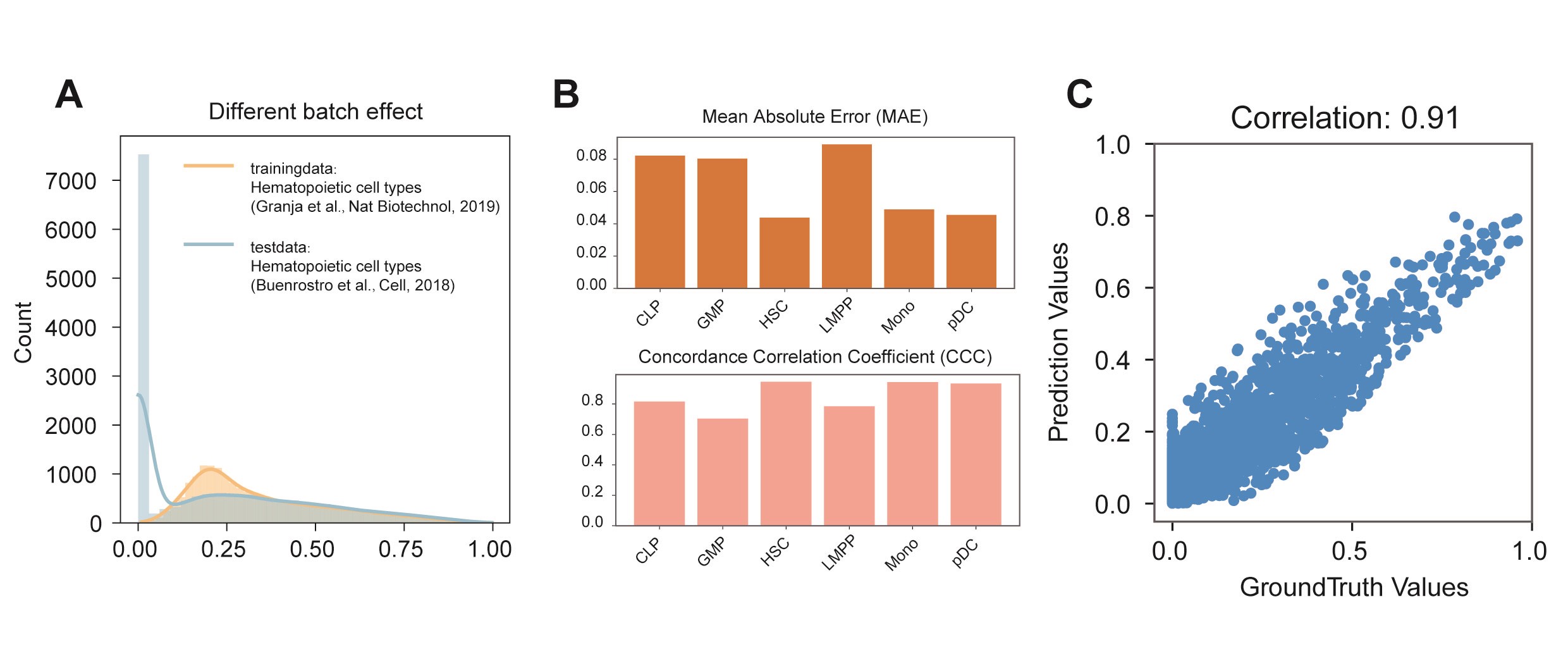

Supplement: supp5_bbaf069 [file supp5_bbaf069.jpeg]

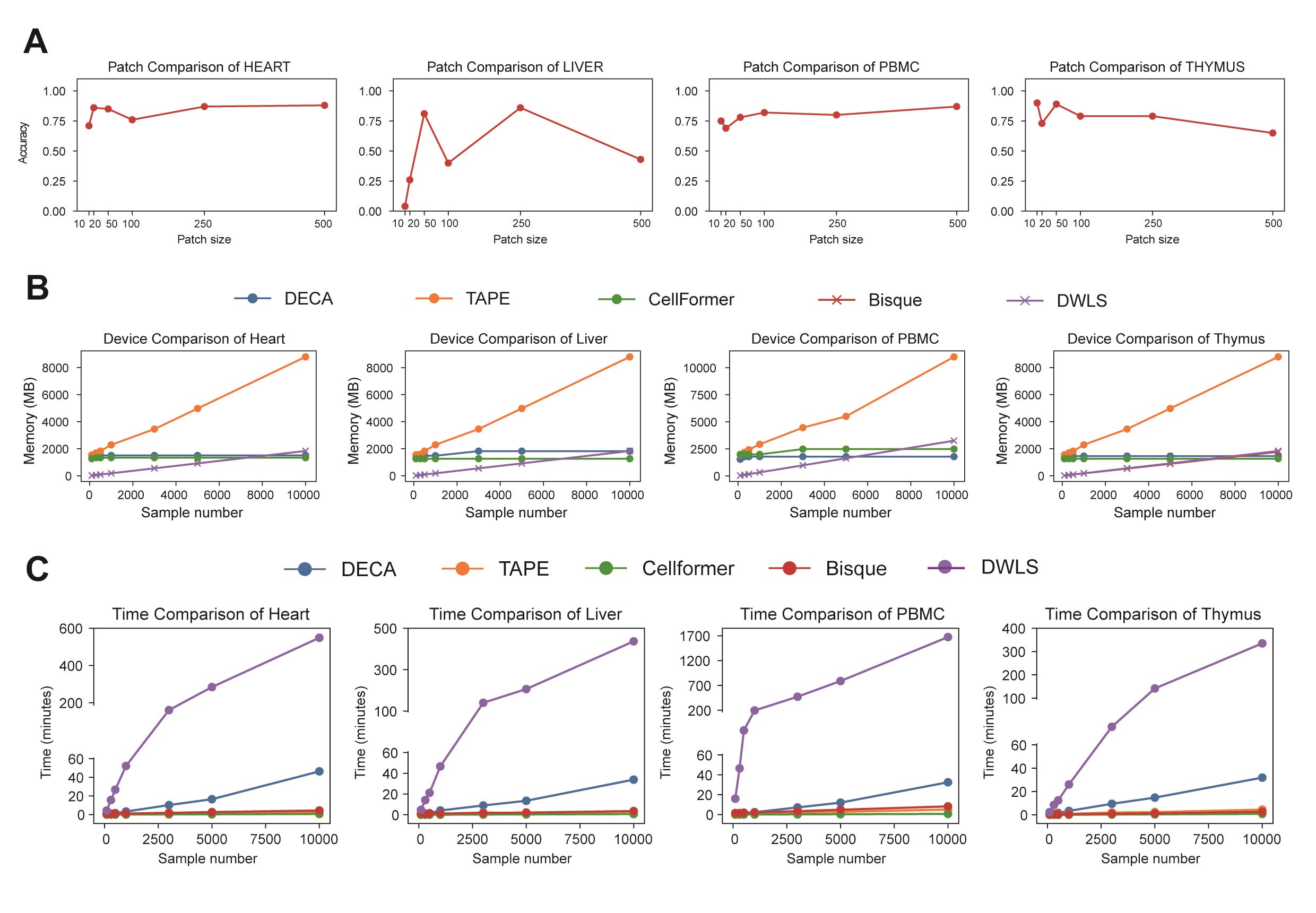

Supplement: supp6_bbaf069 [file supp6_bbaf069.jpeg]

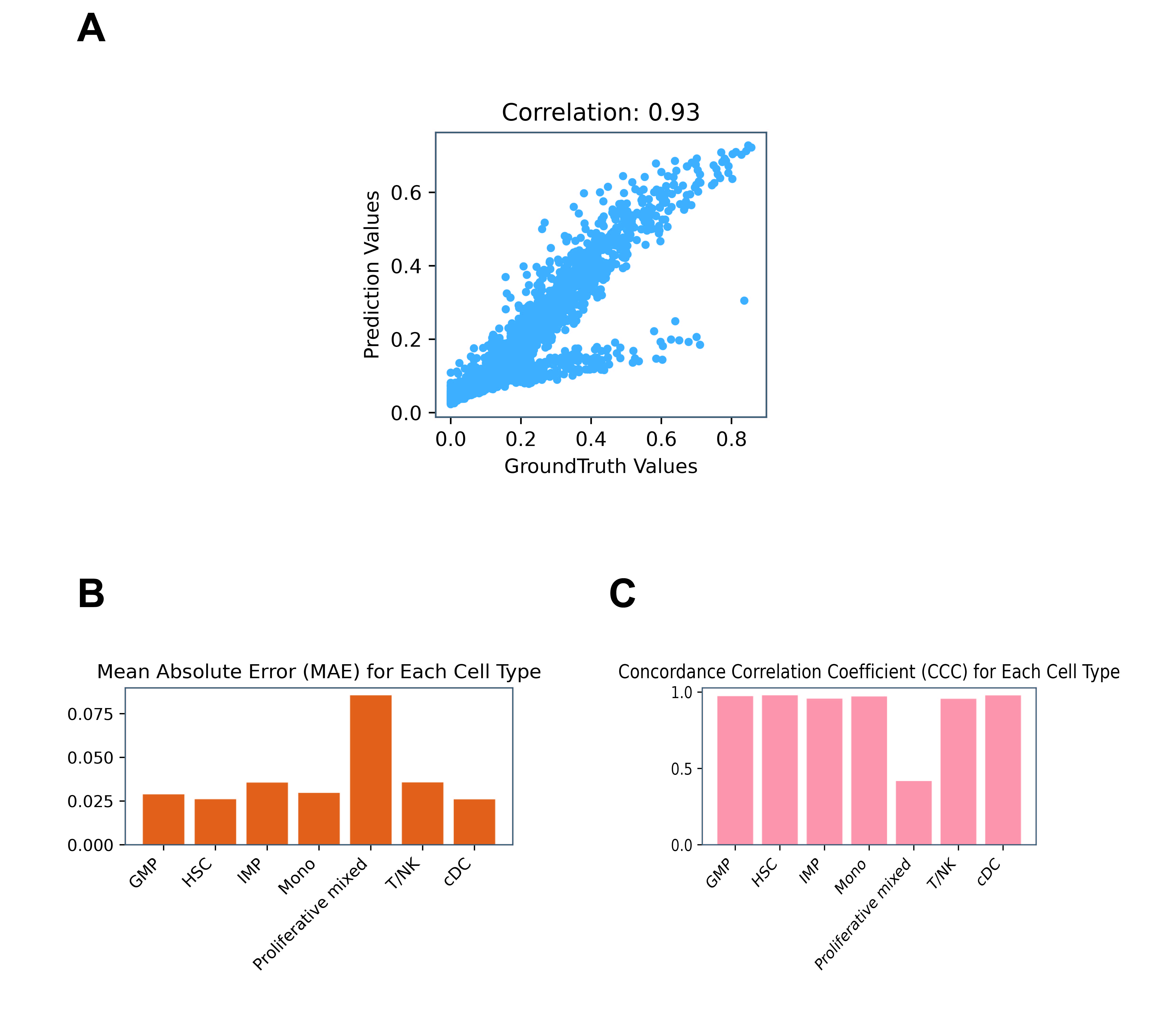

Supplement: supp7_bbaf069 [file supp7_bbaf069.jpeg]

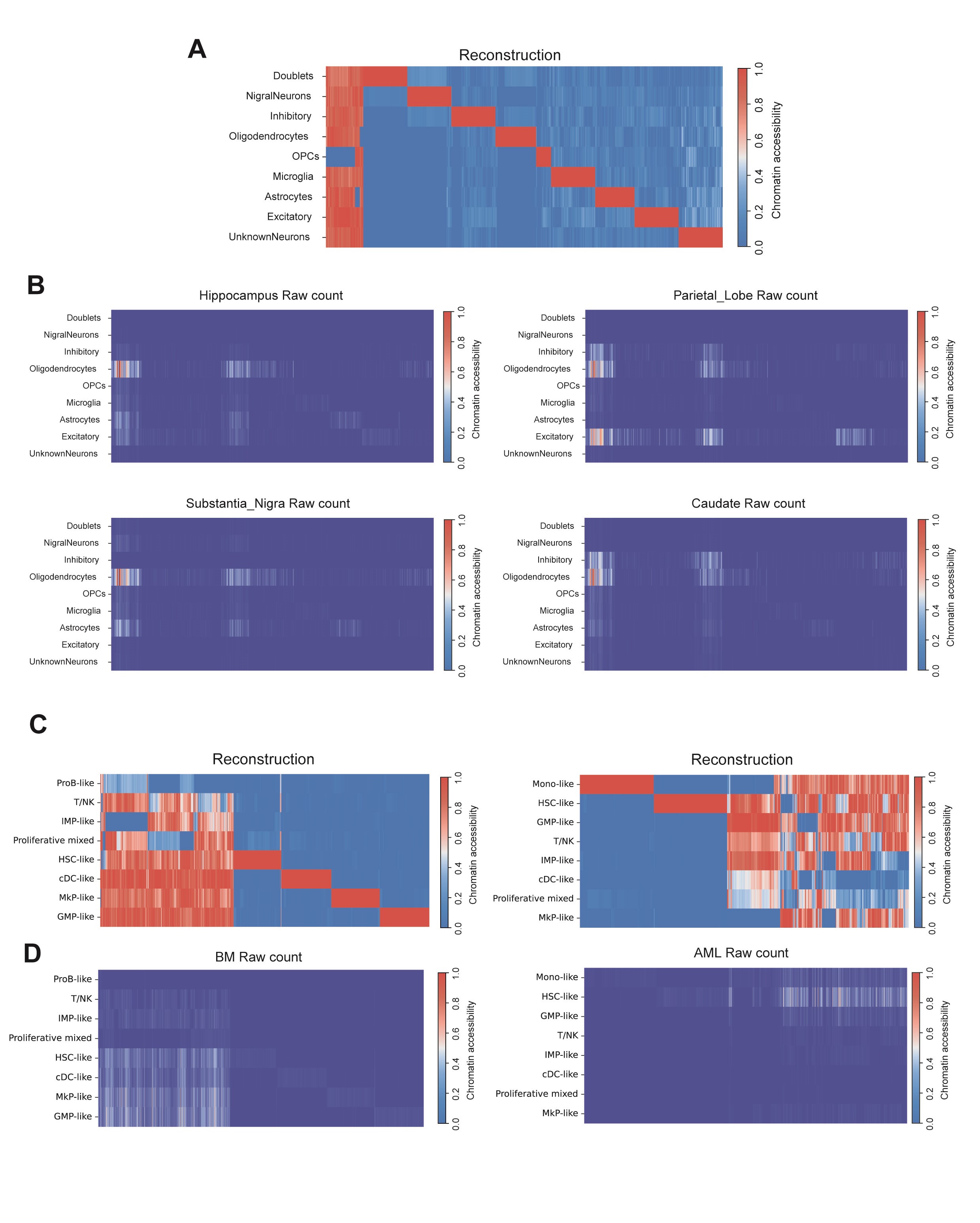

Supplement: supp8_bbaf069 [file supp8_bbaf069.jpeg]

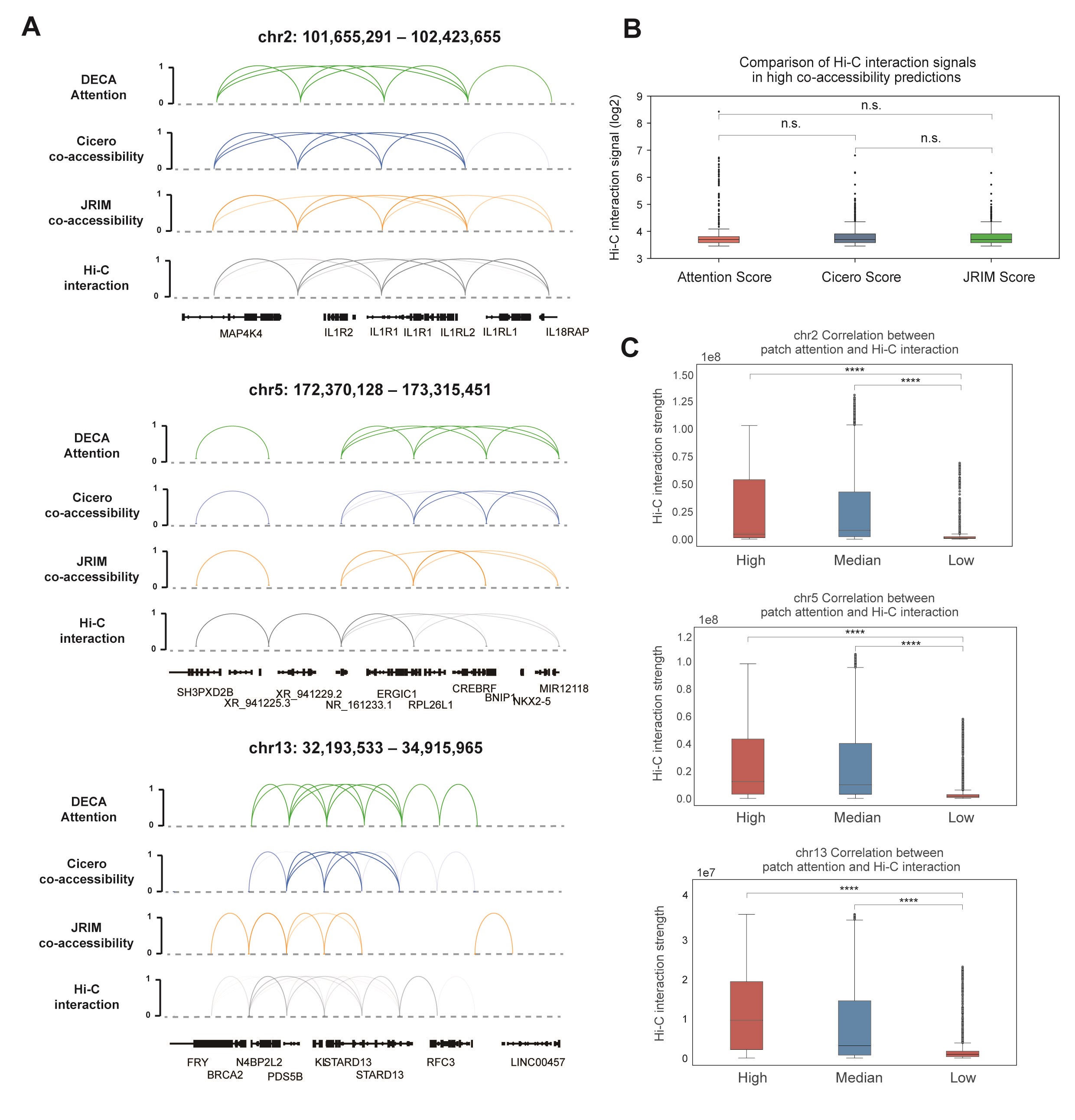

Supplement: supp9_bbaf069 [file supp9_bbaf069.jpeg]

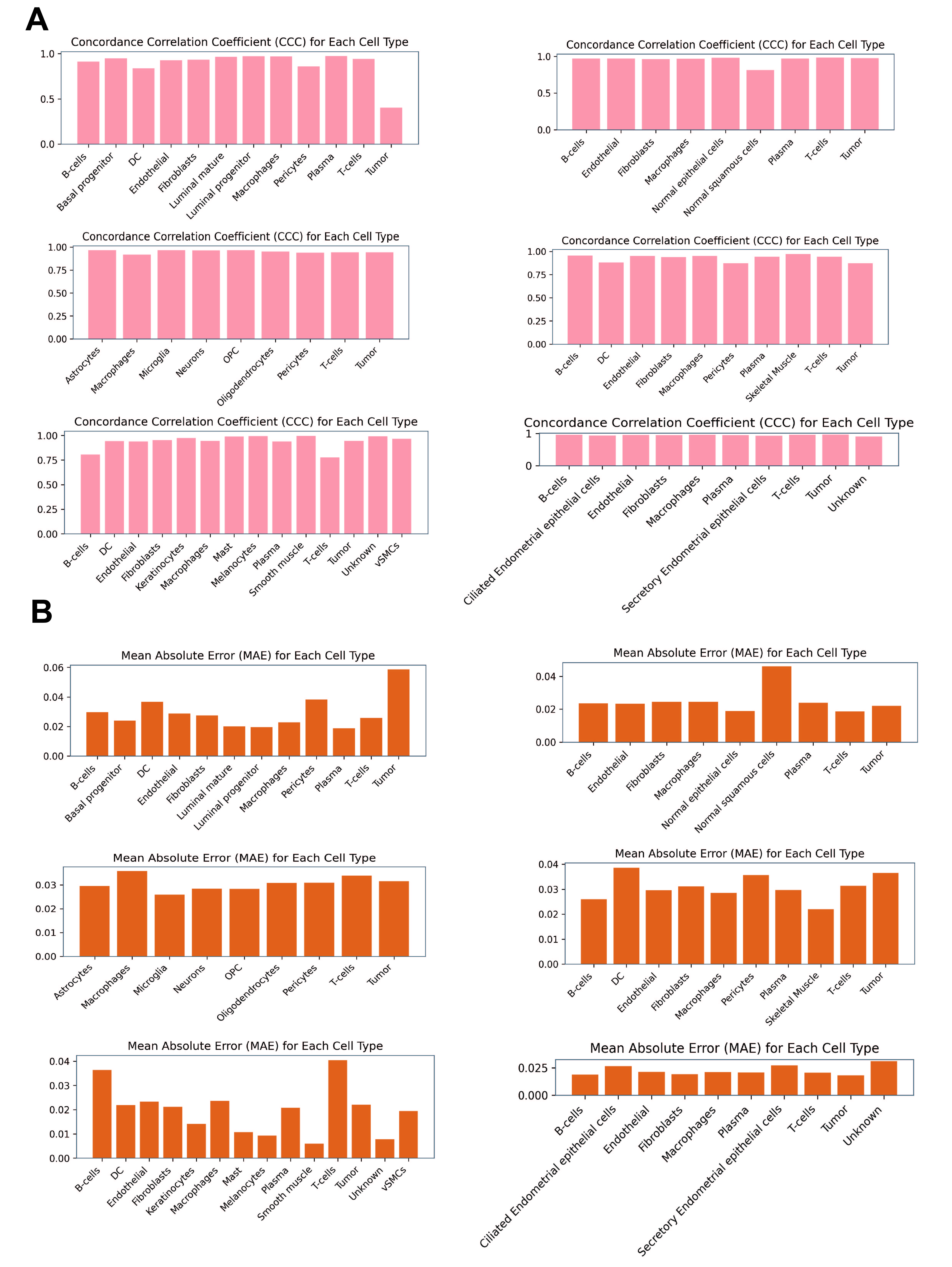

Supplement: supp10_bbaf069 [file supp10_bbaf069.jpeg]

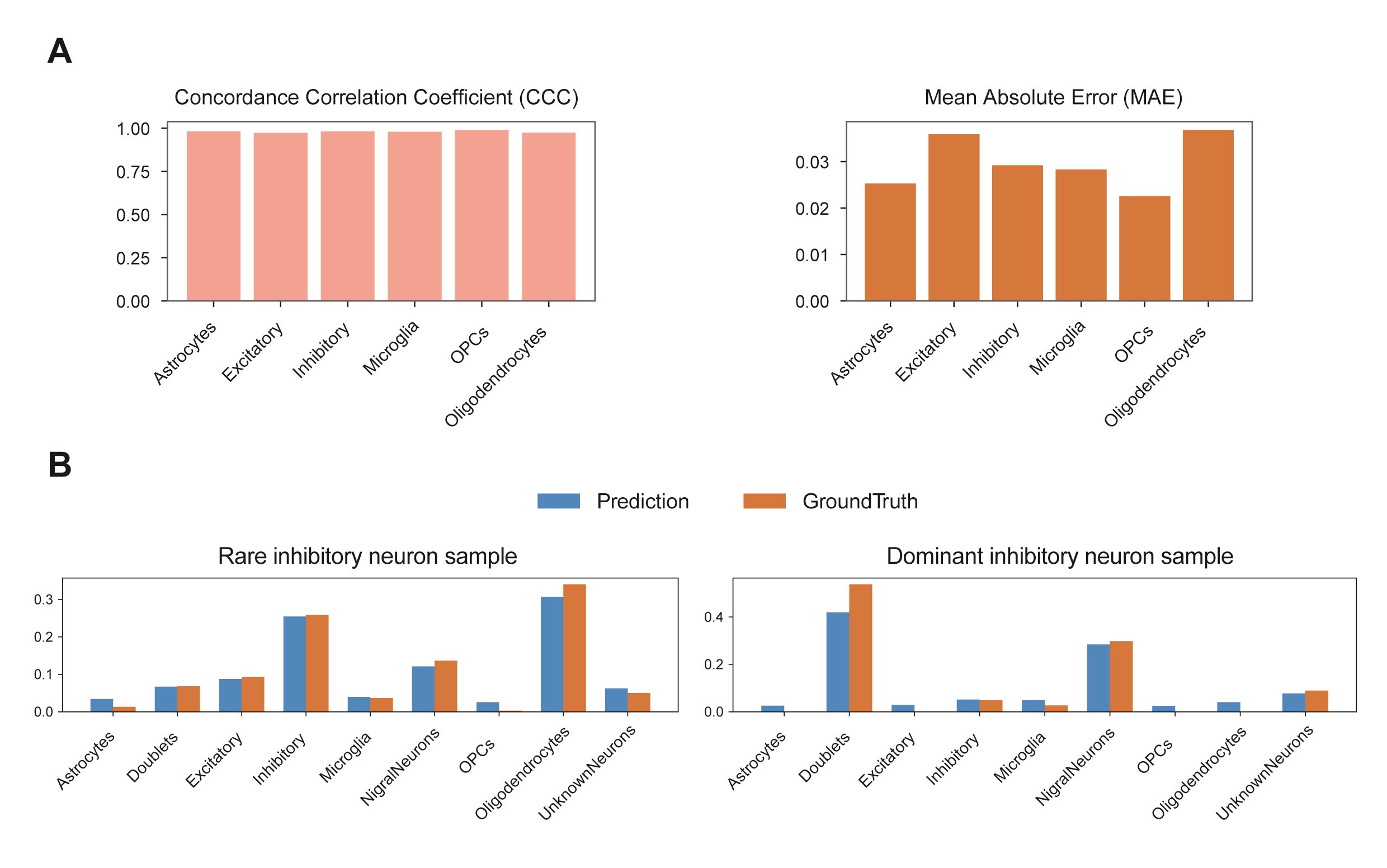

Supplement: supp11_bbaf069 [file supp11_bbaf069.jpeg]
