# Supplementary material for: DECA: harnessing interpretable transformer model for cellular deconvolution of chromatin accessibility profile
Source: Brief Bioinform. 2025 Feb 23;26(1):bbaf069. doi: 10.1093/bib/bbaf069 (PMC11847511; doi:10.1093/bib/bbaf069)
Supplement: Supplementary_Fig_legend_Highlight_0107_bbaf069 [file supplementary_fig_legend_highlight_0107_bbaf069.docx]

**Figure S1 Extract the trained reference from single-cell ATAC data from multiple tissue.**

(**A**-**D**) DECA randomly combined and adjusted numerous cells, each containing precise cell type labels to generate pseudo-bulk.

**Figure S2 Model training details of PBMC datasets.**

(**A**) The box plots showcase the distribution of cell type proportions in the PBMC data, which generation from single-cell ATAC datasets.

(**B**) These bar plots show that the cell type proportions within a single sample were modeled to fit a Dirichlet distribution (the y-axis represents the cell-type proportions, the x-axis represents pseudo-bulk samples).

(**C**) These bar plots display the distribution of normalized chromatin accessibility after preprocessing.

(**D**) These bar plots show that evolution of the loss function across iteration training. The y-axis illustrates the loss values, while the x-axis corresponds to the number of iterations.

**Figure S3 Model training results of PBMC datasets.**

(**A**) The scatter plot representing the correlation of DECA-predicted cell type proportions across all chromosomes using 500 fitted PBMC samples. The *x*-axis represents the cell-type proportion of ground truth, while the *y*-axis represents the predicted proportion.

(**B**) The scatter plot represents correlation within 500 fitted PBMC samples among each cell-type. The *x*-axis represents the accessibility of ground truth, while the *y*-axis represents the predicted accessibility.

**Figure S4 Model training results of PBMC datasets among each chromosome.**

(**A**-**B**) These bar plots illustrate the accuracy of DECA predictions across all chromosome. CCC represents Lin's concordance correlation coefficient, measuring the consistency between predicted scores and ground truth. MAE represents the mean absolute error, assessing the accuracy of predictions. A higher CCC and lower MAE indicate better performance (see **Methods**).

**Revised Figure S5. Control and adjustment of batch effects.**

(**A**) The density plot illustrates the single-cell ATAC-seq data from different batches used for training and testing.

(**B**) Bar plots of DECA accuracy: Lin's concordance correlation coefficient (CCC) and mean absolute error (MAE), where higher CCC and lower MAE indicate better performance.

(**C**) The scatter plot shows the correlation within samples containing hematopoietic cell types. The x-axis represents the cell-type proportion of ground truth, while the y-axis represents the predicted proportion.

**Revised Figure S6. Performance evaluation and benchmarking of DECA across different metrics.**

(**A**) Impact of patch size on DECA's performance. Line plot showing the effect of different patch sizes (10, 20, 50, 100, 250, 500) on DECA's training efficiency and performance.

(**B**) Comparison of memory usage across tools for large-scale datasets. Line plot comparing the memory usage of DECA, TAPE, Cellformer, Bisque, and DWLS (Device) for large-scale datasets.

(**C**) Comparison of runtime across tools for different datasets. Line plot comparing the runtime of DECA, TAPE, Cellformer, Bisque, and DWLS (Time) for datasets with sample sizes ranging from 0 to 10,000.

**Figure S7 Model training results of AML datasets.**

(**A**) The scatter plot represents correlation within 500 fitted AML samples among each chromosome. The *x*-axis represents the cell-type proportion of ground truth, while the *y*-axis represents the predicted proportion.

(**B**-**C**) These bar plots describe the illustrates the accuracy of the DECA. CCC represents Lin's concordance correlation coefficient, measuring the consistency between predicted scores and ground truth. MAE represents the mean absolute error, assessing the accuracy of predictions. A higher CCC and lower MAE indicate better performance (see **Methods**).

**Figure S8 Reconstructed accessibility results from golden benchmark datasets.**

(**A**) The heatmap illustrates the reconstructed accessibility matrix derived from pseudo-bulk samples of the brain tissue.

(**B**) These heatmaps illustrate the raw accessibility matrix extracted from pseudo-bulk samples.

(**C**) These heatmaps illustrate the reconstructed accessibility matrix derived from pseudo-bulk samples of bone marrow (BM) and acute myeloid leukemia (AML) samples.

(**D**) These heatmaps illustrate the raw accessibility matrix extracted from pseudo-bulk samples of BM and AML samples.

**Revised Figure S9. Benchmarking and performance evaluation of patches-attention.**

(**A**) Representative examples of the cis-regulatory interaction network generated by DECA, Cicero, JRIM and Hi-C interactions.

(**B**) Box plots illustrating a comparative analysis of the overlap in Hi-C interaction signals with patch attention, and the high co-accessibility generated by Cicero and JRIM. P-values were calculated using an unpaired two-sample Student's t-test. **P* < 0.05; ***P* < 0.01; ****P* < 0.001; n.s., not significant.

(**C**) These box plots illustrate the correlation between high Hi-C interactions and regions with different levels of patch attention weights on representative chromosome (e.g., chr2, chr5, chr13). P-values were calculated using an unpaired two-sample Student's t-test. **P* < 0.05; ***P* < 0.01; ****P* < 0.001; n.s., not significant.

**Figure S10 Model training results across each cancer.**

(**A**-**B**) These bar plots describe the illustrates the accuracy of the DECA across each cancer. CCC represents Lin's concordance correlation coefficient, measuring the consistency between predicted scores and ground truth. MAE represents the mean absolute error, assessing the accuracy of predictions. A higher CCC and lower MAE indicate better performance (see **Methods**).

**Revised Figure S11. Control and adjustment of inhibitory neuron across brain regions.**

(**A**) Bar plots of DECA accuracy: Lin's concordance correlation coefficient (CCC) and mean absolute error (MAE), where higher CCC and lower MAE indicate better performance.

(**B**) These bar plots show the predicted (blue) vs. ground truth (yellow) proportions. The left plot displays the prediction results for rare inhibitory neurons, while the right plot shows the prediction results for dominant inhibitory neurons.
